# Environmental oxygen regulates astrocyte proliferation to guide angiogenesis during retinal development

**DOI:** 10.1242/dev.199418

**Published:** 2021-05-07

**Authors:** Robin M. Perelli, Matthew L. O'Sullivan, Samantha Zarnick, Jeremy N. Kay

**Affiliations:** 1 Department of Neurobiology, Duke University School of Medicine, Durham, NC 27710, USA; 2 Department of Ophthalmology, Duke University School of Medicine, Durham, NC 27710, USA; 3 Ophthalmology Residency Program, Duke University School of Medicine, Durham, NC 27710, USA; 4 Department of Cell Biology, Duke University School of Medicine, Durham, NC 27710, USA

**Keywords:** Astrocyte, Development, Hypoxia, Oxygen-induced retinopathy, Retina, Retinopathy of prematurity, Mouse

## Abstract

Angiogenesis in the developing mammalian retina requires patterning cues from astrocytes. Developmental disorders of retinal vasculature, such as retinopathy of prematurity (ROP), involve arrest or mispatterning of angiogenesis. Whether these vascular pathologies involve astrocyte dysfunction remains untested. Here, we demonstrate that the major risk factor for ROP – transient neonatal exposure to excess oxygen – disrupts formation of the angiogenic astrocyte template. Exposing newborn mice to elevated oxygen (75%) suppressed astrocyte proliferation, whereas return to room air (21% oxygen) at postnatal day 4 triggered extensive proliferation, massively increasing astrocyte numbers and disturbing their spatial patterning prior to the arrival of developing vasculature. Proliferation required astrocytic HIF2α and was also stimulated by direct hypoxia (10% oxygen), suggesting that astrocyte oxygen sensing regulates the number of astrocytes produced during development. Along with astrocyte defects, return to room air also caused vascular defects reminiscent of ROP. Strikingly, these vascular phenotypes were more severe in animals that had larger numbers of excess astrocytes. Together, our findings suggest that fluctuations in environmental oxygen dysregulate molecular pathways controlling astrocyte proliferation, thereby generating excess astrocytes that interfere with retinal angiogenesis.

## Abstract (precis)

**Highlighted Article:** Oxygen regulates proliferation of immature retinal astrocytes. Perturbing this mechanism inflates astrocyte numbers, disrupts retinal angiogenesis and leads to vascular pathologies resembling retinopathy of prematurity.

## INTRODUCTION

Coordination between growing neurons, glia and blood vessels is essential for building a functional nervous system. A striking example of such coordination occurs in the retinal nerve fiber layer (RNFL), where development of retinal ganglion cell (RGC) axons, astrocytes and vasculature is precisely orchestrated to support visual function. Developing astrocytes and vasculature enter the retina at the optic nerve head and spread centrifugally through the RNFL to colonize the entire retinal surface (Selvam et al., 2018). These migrations are coordinated through a sequential series of cell-cell interactions: RGC axons guide astrocyte migration, and astrocytes in turn guide endothelial cell growth (Dorrell et al., 2002; Fruttiger et al., 1996; Gerhardt et al., 2003; O'Sullivan et al., 2017). Perturbation of these interactions leads to disruption or arrest of angiogenesis (Duan and Fong, 2019; Fruttiger et al., 1996; O'Sullivan et al., 2017; Tao and Zhang, 2016), which is a hallmark of human retinal developmental vascular disorders, such as retinopathy of prematurity (ROP) (Foos, 1987; Hellström et al., 2013). It is therefore of great interest to understand the developmental mechanisms that enable orderly progression of retinal angiogenesis and how these mechanisms might become perturbed in the context of ROP pathology.

The principal risk factors for ROP are prematurity, low birth weight, and supplemental oxygen. In infants with ROP, halted extension of nascent vessels leaves the peripheral retina avascular (Foos, 1987). This is commonly termed Phase I of ROP. Subsequently, in Phase II, hypoxia in the ischemic peripheral retina stimulates neovascularization, which can lead to retinal detachment and vision loss (Hellström et al., 2013). Mechanistically, exposure to high oxygen levels is thought to provoke ROP by diminishing the molecular drive for angiogenesis. Although hyperoxia could explain the wavefront arrest, it remains unclear why vessels do not resume their orderly progression through the RNFL once normoxic conditions are restored.

One possible explanation for this failure of wavefront progression is that the astrocytic template for angiogenesis becomes disturbed. In normal development, astrocytes arrange their somata and arbors into a pre-pattern resembling a capillary bed. This astrocyte template is required for angiogenesis and exerts powerful effects on the patterning of the vasculature (Fruttiger et al., 1996; Gerhardt et al., 2003; O'Sullivan et al., 2017; Tao and Zhang, 2016). Despite this well-established astrocytic function, the role of astrocytes in ROP remains obscure. As in mice, human astrocytes also migrate ahead of the vasculature (Chan-Ling et al., 2004). ROP histopathological studies showed that astrocytes are the major cell type within the fibrovascular ridge – a pathological structure that forms at the site where vessel growth stalls. Moreover, astrocytes are rarely detected peripheral to this ridge (Sun et al., 2010). These observations suggest that astrocyte patterning might be disturbed in ROP. However, despite some hints from animal studies (Duan et al., 2017; Morita et al., 2016; Zhang et al., 1999), it is unknown whether astrocyte development is impacted by ROP risk factors, such as elevated oxygen. Resolving this issue could reveal new developmental mechanisms regulating normal astrocyte development, and would clarify whether altered astrocyte development underlies ROP vascular pathology.

To investigate these questions, we sought to understand astrocyte development in a mouse model of oxygen-induced retinopathy (OIR). In the most common model, mouse pups are exposed to 75% O_2_ from postnatal day (P) 7 to P12 (Smith et al., 1994). This manipulation causes central capillary obliteration during the period of high oxygen, followed by neovascularization upon return to room air. Although this model is powerful for exploring mechanisms of neovascularization and oxygen toxicity, it has some limitations as a model of ROP. First, the phenotype of mouse OIR – central vaso-obliteration and neovascularization – does not resemble the peripheral avascularity seen in ROP, nor the hyperplasia at the vascular-avascular junction (Foos, 1987). Second, the oxygen manipulation in OIR happens at a later ontogenetic stage than in ROP – too late to influence most of the key events in primary angiogenesis. Specifically, the majority of astrocyte differentiation has already occurred by P7, and the primary vascular plexus has already been established. For these reasons, we employed a protocol in which oxygen exposure starts at earlier stages. Unlike in standard OIR, a period of high oxygen at P0 can cause long-term retinal vascular pathology with features reminiscent of advanced ROP (Lajko et al., 2016; McMenamin et al., 2016). Thus, mouse models featuring early oxygen elevation may have utility in understanding ROP pathogenesis and astrocyte involvement.

Here, we show that neonatal exposure to 75% O_2_ has long-lasting consequences for development of RNFL astrocytes and vasculature. In our neonatal oxygen-induced retinopathy (NOIR) model, mice were raised in high O_2_ conditions starting at P0 and returned to room air at P4. Subsequently, NOIR mice develop vitreous hemorrhages and persistent retinal degenerative changes. To understand the origin of these defects, we examined development of vasculature and astrocytes in the NOIR model. We found that return to normoxia triggers a surge in astrocyte mitotic activity producing a vast excess of astrocytes at an age when the astrocyte population is normally declining (Puñal et al., 2019). Environmental hypoxia (10% O_2_) similarly stimulates astrocyte proliferation, suggesting that a relative decrease in oxygen is the mitogenic stimulus. Astrocyte proliferation in the NOIR paradigm was blunted by cell type-specific knockout of HIF2α (also known as EPAS1), indicating that astrocyte-intrinsic oxygen sensing regulates their mitotic activity. Following the initial proliferation, the severity of persistent astrocyte defects was variable between animals. Strikingly, the severity of vascular defects was strongly correlated with the severity of astrocyte overproduction. Our results suggest that the number of retinal astrocytes is modulated by oxygen through HIF2α, and that dysregulation of this pathway perturbs formation of the angiogenic astrocyte template leading to defective angiogenesis.

## RESULTS

### Neonatal hyperoxia disrupts retinal vascular development

**Fig. 1.**
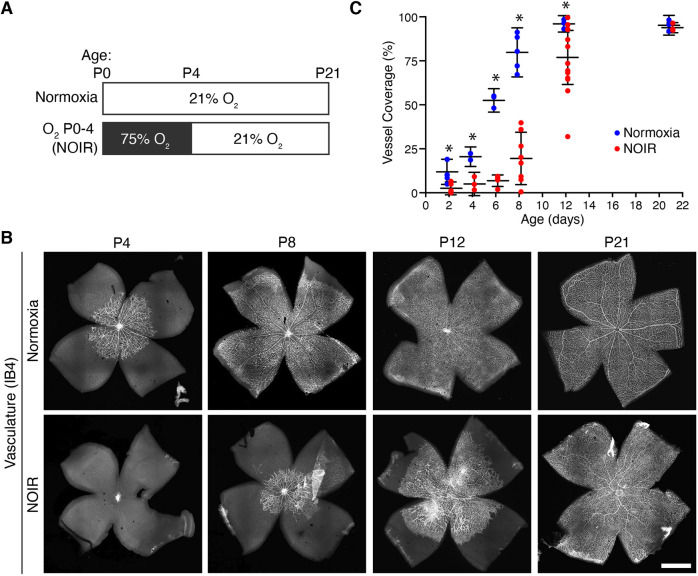
**Excess oxygen prevents retinal angiogenesis.** (A) NOIR protocol timeline. Control (top) and experimental (bottom) animals were exposed to room air (21% O_2_) or high-O_2_ (75%) at the indicated ages. (B,C) Vascular development is delayed by the NOIR protocol. (B) Representative confocal images of vasculature in IB4-stained flat-mounted retinas. Note the absence of vasculature at P4 in NOIR animals. Scale bar: 1 mm. (C) Quantification of vascularized retinal area across development. Angiogenesis was significantly delayed in NOIR animals. Two-way ANOVA: Main effect of age *F*(5,50)=82.6, *P*<0.0001; main effect of oxygen *F*(1,50)=55.7, *P*<0.0001; interaction, *F*(5,50)=9.4, *P*<0.0001. *P<0.05 by Holm–Sidak multiple comparisons test. Error bars, mean±s.d.

To investigate how early exposure to excess oxygen affects retinal vascular development, we raised newborn CD-1 mice in high O_2_ (75%) or room air (21% O_2_) from P0 to P4 (Fig. 1A). Vessel development was assessed during and after the high-O_2_ period (P4-P21) by staining flat-mounted retinas with the endothelial cell marker isolectin B4 (IB4), and quantifying the retinal area covered by vessels. In control mice, centrifugally advancing vessels reached the retinal periphery between P8 and P12 (Fig. 1B,C). By contrast, angiogenesis was significantly delayed in the high-O_2_ group (Fig. 1B,C). Consistent with prior reports (Morita et al., 2016; West et al., 2005), high O_2_ prevented vascular sprouting from the optic nerve head (ONH) with the rare exception of a few stray vessels escaping into the proximal retina. Only after mice were returned to normoxic conditions did the primary vascular plexus begin to grow. During this period, the position of the advancing wavefront was more variable in high-O_2_ mice compared with controls (Fig. 1C; note spread of individual datapoints at P8 and P12). Eventually, however, the vasculature reached the retinal margin in all treated mice, such that the entire RNFL was vascularized by P21 (Fig. 1C).

We next tested whether high O_2_ can also suppress angiogenesis at later stages of its progression. O_2_ exposure starting at P2 or P4, while angiogenesis is underway, caused central capillary loss reminiscent of the widely used mouse OIR paradigm (Fig. S1; Dorrell et al., 2010; O'Bryhim et al., 2012; Ritter et al., 2006), but did not prevent angiogenic sprouting toward the periphery. Thus, O_2_ exposure selectively delays the onset of angiogenesis without delaying its progression.

**Fig. 2.**
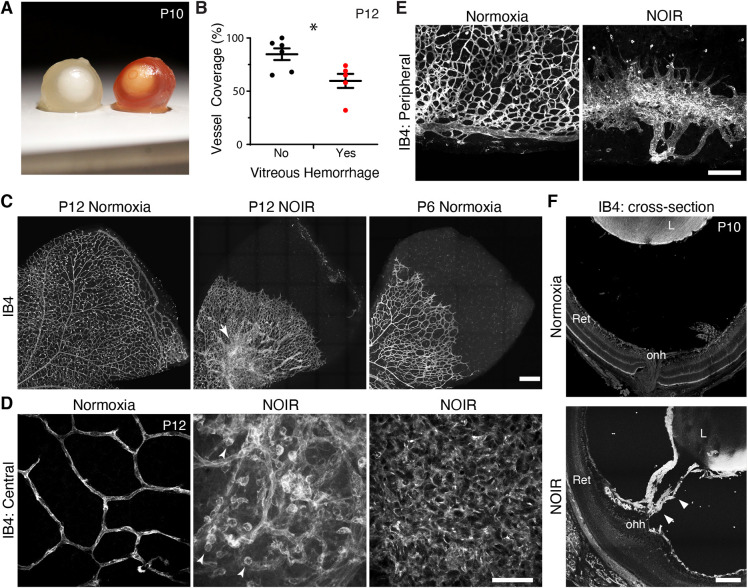
**Angiogenesis is abnormal following return to room air in NOIR protocol.** (A) Gross vitreous hemorrhage was observed in a subset of freshly dissected eyes from NOIR-exposed CD-1 mice (right, hemorrhage; left, normal albino globe). (B) Angiogenesis was more severely delayed in P12 NOIR retinas that exhibited hemorrhage. Two-tailed *t*-test: no hemorrhage, 84.7±5.5% vascular coverage; vitreous hemorrhage, 59.6±6.6% vascular coverage; *P=0.026. (C-E) En-face confocal images of IB4^+^ retinal vasculature, illustrating vascular phenotypes in NOIR-treated mice. (C) Overall vascular organization. Images are oriented with ONH down/left. Note peripheral avascular zone, absence of large radial vessels, and central hyperdensity/irregularity in P12 NOIR example. Arrow indicates vascular clump delaminated from RNFL (also see D, center). Right-hand image shows P6 control with vascular wavefront eccentricity matched to P12 NOIR. NOIR morphology does not resemble normal development. (D) Representative examples of NOIR vascular pathologies in central retina. Left: Orderly capillary patterning in P12 controls. Center: P12 NOIR retina with disorganized RNFL vasculature and a delaminated vascular clump (similar to that indicated by the arrow in C). Round cells (arrowheads) are IB4^+^ macrophages. Right: Lawn of endothelial cells lacking capillary morphology, typical of severe NOIR cases (*n*=5/11 P12 NOIR mice). (E) Vasculature in P12 peripheral retina. Controls (left) show complete primary plexus with terminal circumferential vessel. In NOIR animals (right), retinal periphery was typically ringed by vitreous-derived vascular tissue that lacked connection to intrinsic retinal vessels (*n*=7/11 NOIR mice). (F) Whole-eye cryosections reveal hyaloid vessels (arrowheads) traversing the space between retina (Ret) and lens (L) in NOIR but not control animals. onh, optic nerve head. Scale bars: 250 µm (C); 50 µm (D); 150 µm (E); 100 µm (F). Error bars, mean±s.d.

Close examination of vascular phenotypes in P0-P4 high-O_2_ mice revealed that angiogenesis was dysfunctional as well as delayed. We noted three distinct vascular abnormalities at P10-P12. First, some treated animals had conspicuous vitreous hemorrhages (Fig. 2A,B), which were never seen in controls of any age (*n*=5/11 high-O_2_ mice with hemorrhage; *n*=0/6 in normoxic mice). The presence of vitreous hemorrhage was correlated with the severity of vascular delay at P12 (Fig. 2B). Second, the hyaloid system of high-O_2_-treated eyes failed to regress. Dense hyaloid vessels were evident throughout the vitreous during dissection (all treated animals); the hyaloid artery could be seen emerging from the optic nerve head (Fig. 2F; *n*=2/2 sectioned eyes from separate animals); and vascular tissue originating from the hyaloid vessels was often present circumferentially in the peripheral retina (Fig. 2E). Third, all treated retinas, regardless of vessel coverage delay, exhibited abnormal vascular morphology: the radial organization of large vessels was usually disrupted; capillaries were of abnormally large caliber; and endothelial cells often formed lawns without any evident tubular structure (Fig. 2C,D).

Most P12 treated animals exhibited at least one of the two most striking anatomical phenotypes – the lawn-like pattern (Fig. 2D; *n*=5/11 treated animals) and the peripheral angiogenic hyaloid structures (Fig. 2E; *n*=7/11 treated animals). Even in the few eyes that lacked these strong phenotypes (*n*=3/11), vessel network organization was still haphazard, with an increase in vessel tortuosity and smaller capillary loops (Fig. 2C,D, center panels). These vascular phenotypes were not accompanied by defects in overall retinal growth, suggesting that the high-O_2_ treatment selectively perturbed vasculature rather than causing generalized development defects (Fig. S2). A similar phenotype was observed in C57BL/6J mice treated in the same manner (Fig. S3). Together, these findings demonstrate that neonatal hyperoxia not only delays angiogenesis but also causes vascular pathology in the developing mouse retina. Our results therefore establish the P0-P4 high-O_2_ protocol as an experimental model for NOIR.

### High O_2_ suppresses and return to normoxia stimulates astrocyte proliferation

**Fig. 3.**
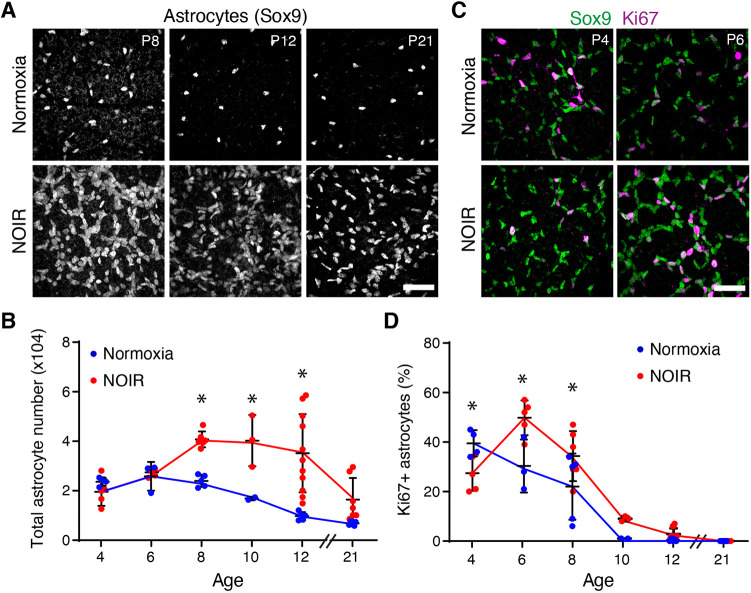
**Astrocyte proliferation is suppressed by high oxygen and stimulated by return to room air.** (A) Representative en-face confocal images of Sox9, an astrocyte nuclear marker. (B) Summary of total astrocyte numbers quantified from images similar to those in A. Astrocyte numbers were significantly higher in NOIR-exposed animals from P8-P12. At P21 there was no group difference but some NOIR animals had nearly triple the number of astrocytes as controls. (C,D) Sox9 and Ki67 double labeling was used to identify mitotically active astrocytes. C shows representative images and D shows quantification of astrocyte proliferation. At P4, proliferation was reduced in NOIR retinas. After return to normoxia, proliferation was increased compared with controls. (B) Two-way ANOVA with Holm–Sidak post-hoc test: main effect of age *F*(5,46)=11.67, *P*<0.0001; main effect of oxygen *F*(1,46)=29.85, *P*<0.0001; interaction, *F*(5,46)=4.999, *P*=0.001. P8, *P=0.082; P10, *P=0.0136; P12, *P<0.0001. (D) Two-way ANOVA with Holm–Sidak post-hoc test: main effect of age *F*(5,49)=74.5, *P*<0.0001; main effect of oxygen *F*(1,49)=7.9, *P*=0.007; interaction, *F*(5,49)=6.336, *P*=0.0001. P4, *P=0.0268; P6, *P=0.0007; P8, *P=0.0122. Scale bars: 50 µm. Error bars, mean±s.d.

We next used the NOIR protocol to examine the effects of excess oxygen on developing astrocytes. The high-O_2_ phase of the protocol, i.e. P0-P4, corresponds to the period when astrocytes are normally migrating to colonize the retinal periphery (Chan-Ling et al., 2009; Fruttiger, 2002; O'Sullivan et al., 2017). Staining for Sox9, an astrocyte nuclear marker, revealed that astrocyte migration was unaffected by hyperoxia. Astrocytes spread outward from the ONH to cover the retina, associated with RGC axons, and became polarized along the centrifugal axis similarly in normoxic and hyperoxic conditions. Accordingly, total astrocyte number was normal in treated mice at the conclusion of the hyperoxic phase (Fig. 3B). Thus, angiogenesis delay (Fig. 2) was not due to impairment in astrocyte colonization of the retina.

Although the astrocyte template was largely unaffected by high O_2_, we found that the hypoxic stress associated with return to room air significantly perturbed astrocyte development. Whole-mount staining for astrocyte nuclear markers revealed that, 4 days after return to room air, treated mice exhibited a striking increase in astrocyte numbers (Fig. 3A). This effect was seen both in CD-1 mice (Fig. 3A,B) as well as the C57BL/6J strain (Fig. S3). To investigate the mechanisms underlying this increase in astrocyte numbers, cohorts of CD-1 pups were exposed either to room air or high O_2_ from P0 to P4 and were collected at varying times thereafter for astrocyte quantification. In wild-type mice, astrocyte numbers increase until P5-6 due to ongoing migration and proliferation; subsequently, their numbers decline substantially due to cell death (Bucher et al., 2013; Chan-Ling et al., 2009; Puñal et al., 2019). A similar pattern was observed in our control normoxia mice (Fig. 3B), but in NOIR mice the number of astrocytes began to increase after P6 (Fig. 3A,B). At P12, treated animals had on average 3.6-fold more astrocytes than normoxic controls, with some animals showing even more dramatic effects (Fig. 3B). Astrocyte numbers remained elevated for weeks, in some cases until at least P21 (Fig. 3B).

Given these large differences in astrocyte numbers, we surmised that astrocyte mitotic activity might be regulated by oxygen. To test this idea, we used Ki67 as an immunohistochemical marker of proliferating cells (Fig. 3C; Fig. S3). In normoxic controls, the fraction of Sox9^+^Ki67^+^ proliferative astrocytes decreased monotonically over development, such that virtually all astrocytes were quiescent by P10 (Fig. 3D). By contrast, in mice exposed to the NOIR protocol, astrocyte proliferation was regulated in a triphasic manner. Initially, during the high-O_2_ phase, astrocyte proliferation was suppressed relative to controls. Then, upon return to room air, there was more proliferation at P6 and P8 before mitotic activity ultimately fell to control levels (Fig. 3C,D). Importantly, the increase in proliferative astrocytes at P6 preceded the increase in astrocyte number at P8, suggesting that proliferation can account for the expansion of the astrocyte population. These findings strongly suggest that return to room air at P4 stimulates astrocyte proliferation, thereby elevating astrocyte numbers in NOIR mice. The proliferative response appears to be reliable across mice, as both the surge in proliferation at P6 (Fig. 3D) and the surge in astrocyte abundance at P8 (Fig. 3B) were highly consistent between animals. Subsequent factors affecting astrocyte number, by contrast, varied substantially between individual mice given the wide range in astrocyte numbers that emerges by P12 (Fig. 3B,D).

### Astrocyte patterning is defective in mice subjected to the NOIR protocol

**Fig. 4.**
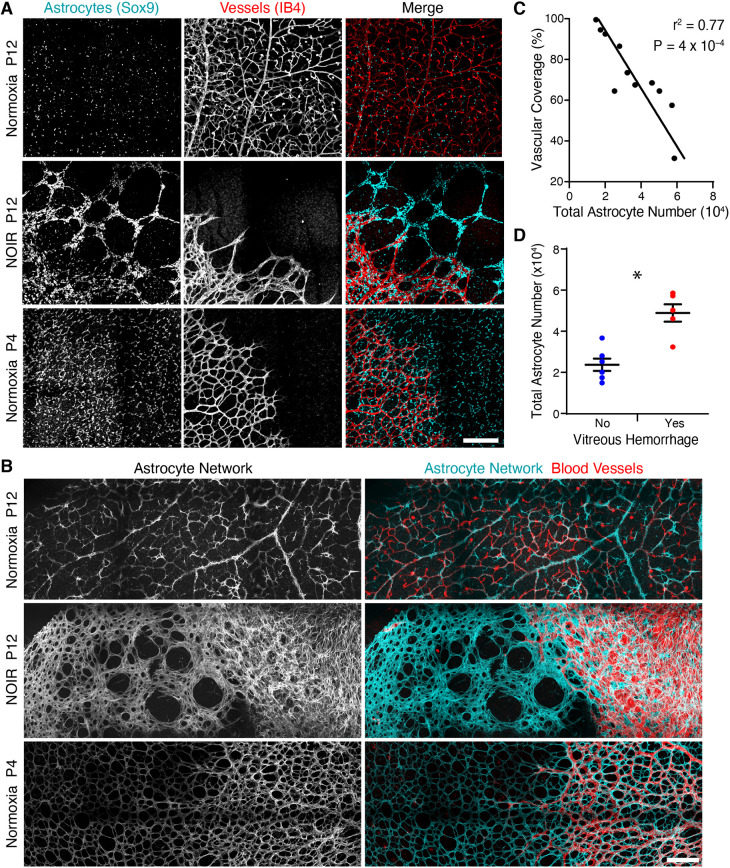
**Astrocyte patterning is abnormal in NOIR mice.** (A,B) En-face confocal images of midperipheral retina stained for astrocyte nuclei (Sox9) and vasculature (IB4 lectin; A), or for astrocyte arbors, labeled by anti-GFAP (P12) or anti-PDGFRα (P4) (B). Control astrocytes and their arbors were homogenously distributed, both in vascularized and avascular retina (A,B). In NOIR retinas, astrocyte somata aggregated in clumps and strings ahead of vascular wavefront (A) generating gaps in arbor coverage (B). Gap size varied across NOIR animals but most (*n*=5/9) exhibited ≥100 µm diameter gaps as shown (A,B). Advancing endothelial cells (red) remained associated with mispatterned astrocytes (A,B). (C) In P12 NOIR animals, retinal vascular coverage is inversely correlated with total astrocyte number. Line slope deviated significantly from zero (*F*=29.53; DFn,d=1, 9; *P*=0.0004). (D) NOIR retinas with vitreous hemorrhage (e.g. Fig. 2A) have significantly more astrocytes than NOIR retinas without hemorrhage. Two-tailed *t*-test: no hemorrhage, 23,721±2982 total astrocytes, *n*=6; vitreous hemorrhage, 48,895±4220, *n*=5; *P=0.0015. Scale bars: 200 µm. Error bars, mean±s.d.

We next tested whether increased astrocyte numbers in NOIR mice are accompanied by changes in astrocyte patterning. Soma positioning and arbor anatomy were evaluated in retinal whole-mounts, using antibodies that label the nucleus (Sox9) or arbors (GFAP, PDGFRα) of developing astrocytes. At P12, astrocyte patterning was abnormal in all NOIR-exposed animals (*n*=11). Behind the angiogenic wavefront, astrocyte arbors were abnormally dense (Fig. 4B), consistent with the cell number increase noted above (Fig. 3). Ahead of the vascular wavefront, astrocytes were arranged into irregular clumps and strands instead of being evenly spaced (Fig. 4A; *n*=9/11 NOIR mice; the other two NOIR mice had completed angiogenesis by P12 so anatomy ahead of the wavefront could not be scored). In the most striking cases (*n*=5/9 scorable NOIR mice), chains of astrocyte somata were arranged into polygons ≥100 µm in diameter (Fig. 4A). This arrangement left large swaths of retinal territory uncovered by astrocytes or their arbors (Fig. 4A,B). Because the pattern of angiogenesis normally follows the astrocyte template, dysmorphia in the astrocyte network may be transmitted to growing vessels. Consistent with this possibility, endothelial tip cells and their filopodia at the vascular wavefront remained strictly colocalized with astrocytes in NOIR mice (Fig. 4B). Together, these findings suggest that oxygen stress alters the astrocyte template in a manner that could delay and/or disrupt angiogenesis.

### Astrocyte number predicts vascular abnormalities

Because neonatal oxygen causes both vascular and glial abnormalities, we investigated whether the vascular defects might in fact originate with the glia. To this end, we exploited the variability in the number of astrocytes in NOIR-exposed retinas at P12 (Fig. 3B). If vessel phenotypes are a consequence of astrocyte phenotypes, we would predict that the NOIR animals with the most severe astrocyte disruptions should also show the most severe vascular pathology. Consistent with this hypothesis, the total number of astrocytes was correlated with the severity of the angiogenesis delay phenotype (Fig. 4C). Furthermore, animals with vitreous hemorrhage had significantly more astrocytes. (Fig. 4D). These observations support the notion that anomalous astrocyte proliferation promotes angiogenic defects in the NOIR model.

### Neonatal oxygen causes enduring retinal abnormalities

**Fig. 5.**
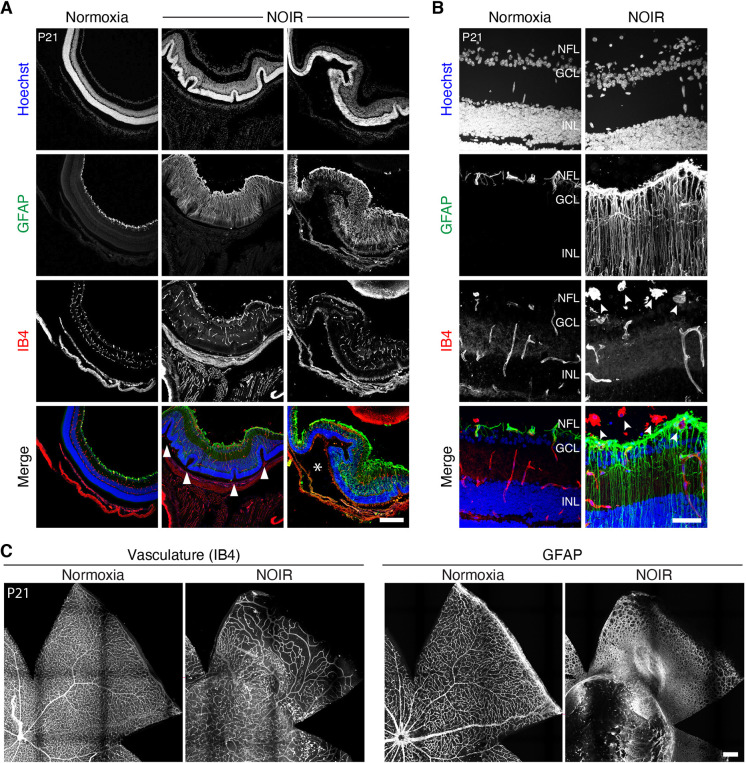
**Neonatal oxygen exposure causes enduring retinopathy.** (A,B) Retinal cross-sections from P21 mice illustrating pathological features of NOIR-exposed retinas. Tissue was stained for vasculature (IB4) and glia (GFAP). (A) Low-magnification images from a normoxic control and two different NOIR-exposed animals. NOIR retinas were thickened relative to controls, with ROP-like outer retinal folding (arrowheads; Foos, 1987) and GFAP throughout the retina indicating reactive Müller gliosis. Images are representative of NOIR phenotypes (*n*=6/7 showed folding; *n*=7/7 showed reactive gliosis). Asterisk marks large subretinal space suggestive of retinal detachment. (B) Higher magnification images illustrate vitreal neovascular clumps in NOIR retinas (arrowheads), and radial GFAP staining by reactive Müller glia. (C) En-face view of P21 retinas illustrating disruptions to vasculature (IB4) and glia (GFAP) that were observed in a majority of NOIR animals (*n*=4/7). GCL, ganglion cell layer; INL, inner nuclear layer; NFL, nerve fiber layer. Scale bars: 250 µm (A,C); 50 µm (B).

To explore the long-term consequences of neonatal exposure to elevated oxygen, we examined NOIR mice and normoxic controls at 3 weeks of age, when the retina is largely mature. Vascular disorganization and persistent hyaloid vessels were still evident in whole-mount NOIR retinas at P21, particularly in cases in which astrocyte numbers remained high (Fig. 5C). To test for other facets of retinal pathology, retinas were cryosectioned and immunostained with vascular and glial markers. High-O_2_ retinas (*n*=7) showed strikingly abnormal cytoarchitecture. In most cases (*n*=6/7), we observed buckling and corrugation of the outer layers (Fig. 5A); this phenotype resembled pathology seen in some advanced ROP samples (Foos, 1987). NOIR retinas also exhibited large-scale reactive gliosis of Müller glia, indicated by upregulation of GFAP (Fig. 5B), consistent with the presence of ongoing tissue stress (*n*=7/7 high-O_2_ eyes). We also noted additional vascular phenotypes in IB4-stained sections that were not evident in the whole-mount preparations (*n*=7/7 eyes). These included laminar disorganization of the intermediate and deep vascular plexuses (Fig. 5A); intravitreal neovascularization (Fig. 5B); and hyperplasic vascular tissue at the inner retinal surface (Fig. 5A). The hyperplasic superficial vasculature was associated with a hyperplasic GFAP^+^ glial network, which likely included excess nerve fiber layer astrocytes as well as the endfeet of reactive Müller glia (Fig. 5B). Together, these observations indicate that a brief period of neonatal hyperoxia during a crucial period of glial-vascular development produces long-lasting deleterious consequences.

### Hypoxia stimulates astrocyte proliferation

**Fig. 6.**
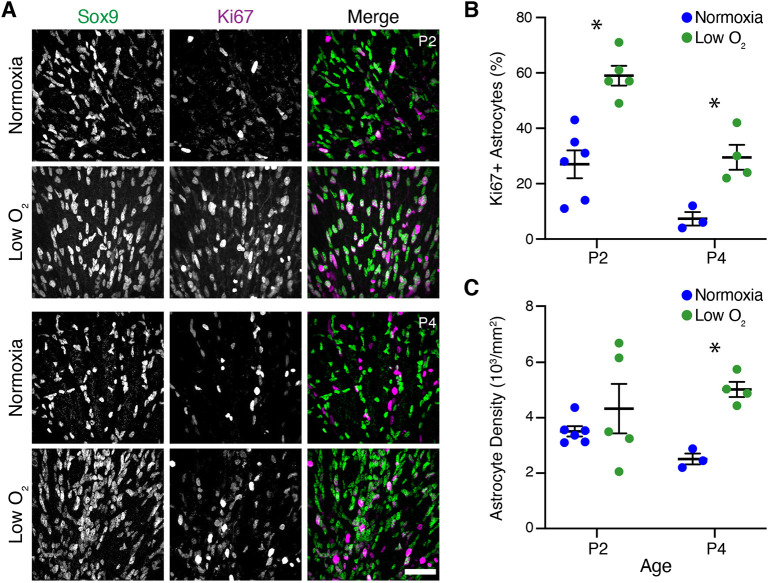
**Hypoxia stimulates astrocyte proliferation.** (A) Representative en-face images showing astrocyte density and proliferation in animals exposed to hypoxia from birth (10% oxygen; low-O_2_ group) or littermate controls (normoxia group). Sox9, astrocytes; Ki67, proliferating cells. All images are from vascularized central retina. (B,C) Effects of low O_2_ on astrocyte abundance (C) and proliferation (B) were quantified from images similar to those in A. More astrocytes were proliferative at P2 and P4 in low-O_2_ mice (B), leading to greater astrocyte density by P4 (C). (B) Two-way ANOVA: main effect of age *F*(1,14)=27.49, *P*=0.0001; main effect of low-O_2_ *F*(1,14)=33.37, *P*<0.0001; interaction, *F*(1,14)=1.10, *P*=0.3121. (C) Two-way ANOVA: main effect of age *F*(1,14)=0.07, *P*=0.7885; main effect of low-O_2_ *F*(1,14)=9.00, *P*=0.0095; interaction, *F*(1,14)=2.32, *P*=0.1502. Significant differences by post-hoc Holm–Sidak test are indicated by asterisks: (B) P2, *P=0.0002; P4, *P=0.0184. (C) P2, *P=0.4434; P4, *P=0.0238. Scale bar: 50 µm. Error bars, mean±s.d.

Because an excess of astrocytes was associated with more severe vascular pathology in NOIR mice, we decided to investigate the mechanisms that lead to astrocyte overproduction. Because astrocyte proliferation was suppressed during the period of high O_2_ and activated upon return to room air (Fig. 3D), we hypothesized that the cellular oxygen-sensing machinery regulates astrocyte proliferation. In this model, the cause of proliferation in the NOIR protocol is relative hypoxia induced by the transition from 75% to 21% oxygen. If this hypothesis is correct, then rearing mice in a hypoxic (low O_2_) environment should mimic the proliferative effects of return to room air in the NOIR protocol. To test this idea, neonatal mice were raised in 10% oxygen from P0 to P4. At both P2 and P4, low-O_2_ mice showed a striking increase in the fraction of Ki67^+^ proliferating astrocytes compared with normoxic littermate controls (Fig. 6A,B). This was accompanied by a corresponding increase in astrocyte abundance: by P4, astrocyte density in low-O_2_ retinas exceeded controls by approximately twofold (Fig. 6C). Therefore, direct hypoxia drives astrocyte proliferation similarly to the room-air phase of the NOIR treatment regime, suggesting that a relative decrement in oxygen availability is the stimulus that drives pathological astrocyte overproduction.

### HIF2α drives astrocyte proliferation in normal development

We next investigated the molecular basis for astrocyte hyperproliferation in NOIR mice. Given the central role of hypoxia in driving mitotic activity (Fig. 6), we focused on the hypoxia-inducible factor (HIF) pathway – a key molecular mechanism for oxygen sensing with known roles in cell proliferation (Hubbi and Semenza, 2015). Retinal astrocytes are likely to be a major site of HIF signaling, as they express high levels of VEGF-A, a direct transcriptional target of HIF transcription factors (Gerhardt et al., 2003; Stone et al., 1995; West et al., 2005). Astrocytic VEGF-A, in turn, is required for initiation of retinal angiogenesis (Rattner et al., 2019). However, it is unclear whether HIF signaling has a direct role in astrocyte development, as previous studies have yielded conflicting results (Duan et al., 2014; Weidemann et al., 2010).

**Fig. 7.**
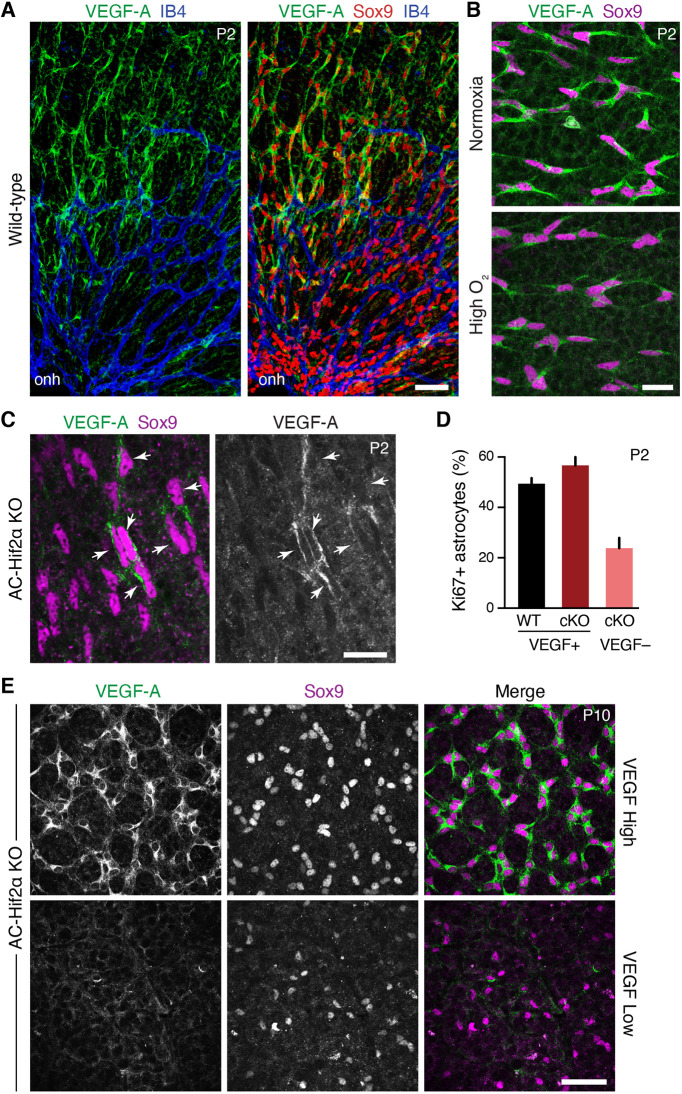
**Astrocyte-specific deletion of Hif2α blocks HIF signaling and impairs astrocyte proliferation.** (A) En-face images of P2 wild-type retinal whole-mounts immunostained for VEGF-A, astrocytes (Sox9) and vasculature (IB4). onh, optic nerve head. Astrocytes are the major VEGF-A^+^ retinal cell type at P2. VEGF-A expression levels are highest in avascular peripheral retina. Also see Fig. S4. (B) Astrocyte VEGF-A expression in mice reared in normoxia (top) or 75% O_2_ (bottom) from P0 to P2. High O_2_ suppresses astrocyte VEGF-A production. (C) In P2 *GFAP-Cre;* Hif2α*^flox/flox^* mice (AC-Hif2α-KO), many astrocytes fail to express VEGF-A, indicating loss of HIF signaling. A subset of astrocytes remains VEGF-A^+^ (arrowheads). (D) Quantification of astrocyte proliferation in wild-type (WT) littermate controls and AC-Hif2α-KO mutant retinas triple stained for Sox9, VEGF-A and Ki67. In mutants, Sox9^+^ astrocytes without HIF signaling (VEGF^–^) are significantly less proliferative than those retaining HIF signaling (VEGF^+^). Mutant VEGF^+^ astrocytes proliferate at a similar rate as WT astrocytes. Error bars: 95% confidence interval. *n*=3 animals and >800 cells analyzed per genotype. (E) Two classes of mutant phenotypes are observed in P10 AC-Hif2α-KO mice. VEGF-high mutants (top) express VEGF-A in all astrocytes; VEGF-low mutants (bottom) lack astrocytic VEGF-A labeling. Note the difference in astrocyte cell density between mutant classes; quantification of astrocyte numbers is provided in Fig. 9C and Fig. S5. Scale bars: 50 µm (A,E); 25 µm (B); 20 µm (C).

To learn whether the HIF pathway might control astrocyte proliferation, we monitored HIF signaling in developing retina by immunostaining for VEGF-A as a readout of HIF activity (Pagès and Pouysségur, 2005). In accordance with past studies (Gerhardt et al., 2003; Morita et al., 2017; Stone et al., 1995; West et al., 2005), we found that VEGF-A was selectively expressed by retinal astrocytes, with the strongest staining in astrocytes of avascular retina ahead of the angiogenic wavefront (Fig. 7A; Fig. S4). Thus, HIF signaling is strongest in astrocytes that lack access to oxygenated blood from the retinal vasculature. We next assessed HIF activity in mice exposed to the NOIR protocol. During the high-O_2_ phase, when astrocyte proliferation is suppressed (Fig. 3D), VEGF-A expression was strongly attenuated (Fig. 7B). On return to room air, however, when astrocytes have become hyperproliferative, astrocytes of avascular retina once again expressed VEGF-A at high levels (Fig. S4C). Together, these findings indicate that local oxygen availability drives astrocyte HIF signaling, establishing this pathway as a candidate to mediate hypoxia-dependent proliferation.

To test the role of HIF signaling in astrocyte proliferation, we used a conditional knockout strategy. Analysis of RNA-seq data (Clark et al., 2019; GSE118614) indicated that HIF2α is the major HIF effector expressed by developing retinal astrocytes, with minimal HIF1α expression. Therefore, we obtained HIF2α-flox conditional mutant mice (Gruber et al., 2007) and crossed them to the astrocyte-specific *GFAP-Cre* strain (O'Sullivan et al., 2017; Puñal et al., 2019; Zhuo et al., 2001). This breeding yielded astrocyte-specific conditional HIF2α knockout animals (abbreviated AC-Hif2α-KO).

Using AC-Hif2α-KO mice, we first investigated the consequences of astrocyte HIF2α deletion for animals raised in normoxia. At P2, the vast majority of wild-type astrocytes (92.1%) were VEGF-A^+^ (*n*=2151 cells from 3 mice). Even though VEGF-A was downregulated in vascularized central retina (Fig. 7A), expression was not yet entirely extinguished – a similar fraction of P2 astrocytes were VEGF^+^ regardless of retinal location (88.7% in vascular central retina; 94.8% in avascular regions). By contrast, in P2 AC-Hif2α-KO mice, a large fraction of retinal astrocytes lacked VEGF-A immunoreactivity (Fig. 7C), suggesting that HIF2α is the major effector of HIF signaling in these cells. A variable subset of AC-Hif2α-KO astrocytes remained VEGF-A^+^ (Fig. 7C), consistent with our previous observations that some astrocytes escape Cre recombination in *GFAP-Cre* mice (O'Sullivan et al., 2017; Puñal et al., 2019). Together, these findings indicate that removal of HIF2α blocks astrocytic HIF pathway activity in a cell-autonomous manner.

To assay astrocyte proliferation in the absence of HIF signaling, we separately quantified Ki67 expression within the two distinct AC-Hif2α-KO astrocyte populations – i.e. VEGF-A^–^ astrocytes that had lost HIF signaling, and VEGF-A^+^ astrocytes in which HIF signaling was intact (Fig. 7C). The fraction of Ki67^+^ proliferating astrocytes in each of these two groups was compared with the fraction observed in wild-type littermate controls. This analysis revealed a significant proliferation phenotype at P2: HIF-deficient astrocytes were less proliferative than VEGF-A^+^ astrocytes from the same retinas, as well as astrocytes from wild-type littermates (Fig. 7D). Impaired proliferation was accompanied by a nearly twofold decrease in mutant astrocyte numbers at P2 (Fig. S5A). Thus, astrocytic Hif2α is required for normal astrocyte proliferation.

**Fig. 8.**
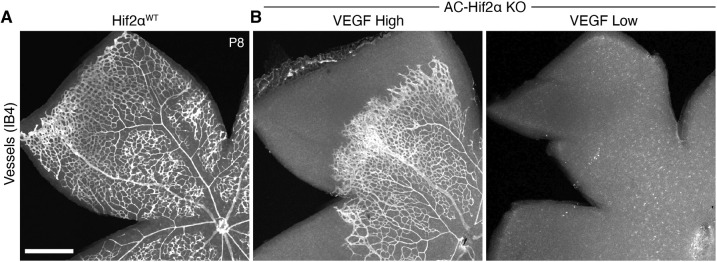
**Angiogenesis is disrupted by HIF2α deletion from astrocytes.** Whole-mount retinas stained with IB4 lectin to reveal vasculature. (A) Primary angiogenesis is complete by P8 in HIF2α^WT^ littermate controls. (B) In AC-HIF2α-KO mutants, angiogenesis is disrupted to varying degrees. VEGF-low mutants (B, right) entirely lack retinal vasculature; remaining punctate signal is from IB4^+^ microglia/macrophages. In VEGF-high mutants (B, left), angiogenesis is delayed in the majority of animals (*n*=7/13) despite preservation of astrocytic HIF2α function (see Fig. 7E). Scale bar: 500 µm.

We next assessed the consequences of astrocyte Hif2α deletion at later developmental stages. Staining for VEGF-A, astrocytes and vasculature revealed two distinct classes of AC-Hif2α-KO mutants during the second postnatal week (Fig. 7E; Fig. S5C,D). The first class of mutants replicated the findings of a previous study by Duan et al. (2014): Astrocyte numbers were reduced (Fig. 7E; Fig. S5C) and retinal vasculature was completely absent (Fig. 8). In this class of mutants, which we denote ‘VEGF-low’, astrocyte VEGF-A staining was almost entirely eliminated, demonstrating effective disruption of HIF signaling (Fig. 7E; Fig. S6). By contrast, astrocyte HIF signaling appeared largely intact in the second class of AC-Hif2α-KO mutants, which we denote ‘VEGF-high’ because virtually all astrocytes continued to express VEGF-A (Fig. 7E). Unlike VEGF-low mutants, retinal phenotypes in VEGF-high mutants were mild: astrocyte numbers were similar to those of wild type (Fig. S5C,D), and retinal vasculature was present and relatively normal, albeit frequently delayed in its development (Fig. 8; *n*=7/13 VEGF-high mutants were delayed relative to wild-type littermates). The all-or-none pattern of VEGF-A expression in P8-P10 mutants was surprising, because in P2 mutants we always observed a mixture of VEGF-A^+^ and VEGF-A^−^ astrocytes (Fig. 7C; Fig. S5B). Given the difference in proliferative capacity between VEGF-A^+^ and VEGF-A^−^ astrocytes at P2 (Fig. 7D), the VEGF-high phenotype is consistent with a model in which VEGF-A^+^ astrocytes may in some cases outcompete their mutant neighbors to take over the entire retina. Therefore, the phenotypes observed in AC-Hif2α-KO mutants together support the conclusion that HIF signaling is required to support astrocyte proliferation during normal retinal development.

### Excessive astrocyte proliferation in the NOIR model requires HIF2α

**Fig. 9.**
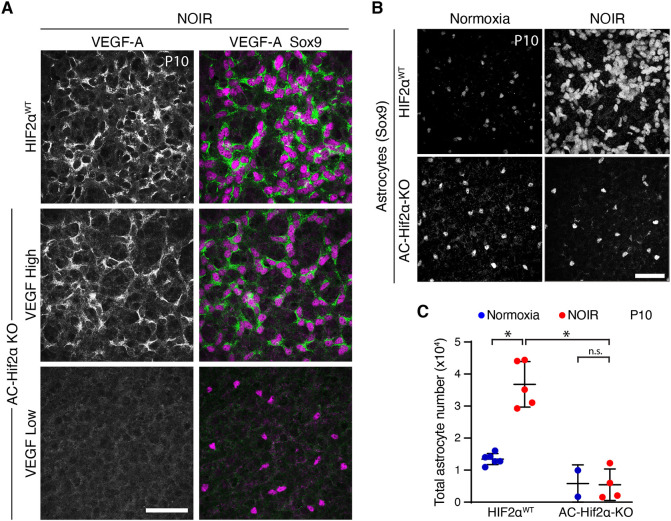
**Astrocyte HIF2α is required for proliferation on return to room air in NOIR model.** (A) HIF pathway activation, evaluated by anti-VEGF-A staining, in retinas from NOIR-exposed AC-Hif2α-KO mutants and HIF2α^WT^ littermate controls. All images are from the outer vascular wavefront. In HIF2α^WT^ controls (top) and mutants of VEGF-high class (center), VEGF-A was expressed by virtually all Sox9^+^ astrocytes, indicating intact HIF signaling. VEGF-low mutants (bottom) lacked VEGF-A expression, indicating successful abrogation of HIF signaling. Also see Fig. S6. (B,C) Effects of the NOIR protocol on P10 astrocyte numbers in AC-Hif2α-KO mice. B shows representative Sox9 images and C shows quantification of total Sox9^+^ astrocytes. NOIR exposure greatly increased astrocyte numbers in HIF2α^WT^ controls, but not in AC-Hif2α-KO mutants. Also see Fig. S5C,D. Two-way ANOVA: main effect of O_2_ treatment *F*(1,13)=19.0, *P*=0.0008; main effect of genotype *F*(1,13)=54.7, *P*=5.2×10^−6^; interaction, *F*(1,13)=20.2, *P*=0.0006. Significant differences by post-hoc Holm–Sidak test are indicated by asterisks: WT normoxia versus hyperoxia, *P=1.7×10^−5^; WT hyperoxia versus KO hyperoxia, *P=2.3×10^−6^; KO normoxia versus KO hyperoxia, *P=0.9354. Scale bars: 50 µm. Error bars, mean±s.d.

Finally, to test whether astrocyte HIF function mediates pathological oxygen-induced proliferation AC-Hif2α-KO mice and littermate controls were subjected to the NOIR treatment regime. Controls included both HIF2α*^flox/flox^* mice lacking the Cre transgene and *GFAP-Cre;* HIF2α*^+/+^* mice, which were phenotypically indistinguishable; together, we refer to them as HIF2α*^WT^* animals. AC-Hif2α-KO mice exhibiting the VEGF-high phenotype (as described above) were excluded from the analysis; it was clear from the VEGF-A staining pattern that this class of mutants did not lack astrocytic HIF2α function (Fig. 9A). Exposure of HIF2α*^WT^* mice to the NOIR protocol induced vascular and astrocyte phenotypes resembling those observed in CD-1 and C57Bl/6J strains (Fig. 9; Fig. S6). This included a dramatic increase in astrocyte numbers by P10 (Fig. 9C). By contrast, no such increase was observed in NOIR-exposed AC-Hif2α-KO mutants (Fig. 9B,C; Fig. S5C,D). These data strongly suggest that HIF2α is required within astrocytes to drive the proliferative response observed following return to room air at P4.

## DISCUSSION

In this study, we found that oxygen stress perturbs development of retinal astrocytes. Return to room air following a limited period of neonatal hyperoxia stimulated exuberant astrocyte mitotic activity, at an age when maturing astrocytes normally cease proliferating. Exposure to neonatal oxygen also perturbed retinal angiogenesis, leading to vascular pathologies. Moreover, the severity of vasculopathy was correlated with total astrocyte number, suggesting that the two phenotypes may be linked. Our results can be explained by the following model. Neonatal hyperoxia recalibrates the baseline oxygen level sensed by astrocytes, such that return to room air triggers a HIF2α-dependent hypoxic response that includes astrocyte proliferation. Subsequently, excessive astrocyte numbers contribute to establishment of developmental vasculopathies. These include delays in peripheral vessel extension; persistent hyaloid vasculature; irregular and abnormally dense endothelial networks lacking regular capillary morphology; and vitreous hemorrhage. Because many of these vascular phenotypes resemble ROP (Eller et al., 1987; Foos, 1987; McMenamin et al., 2016), our results raise the possibility that astrocytes may have an important role in the pathobiology of ROP and related disorders.

### Retinal astrocyte proliferation is bidirectionally regulated by environmental oxygen

It has long been clear that retinal astrocytes are sensitive to tissue oxygenation, as their gene expression patterns and developmental maturation are regulated by access to vasculature (Chan-Ling et al., 2009; Duan et al., 2017; Gerhardt et al., 2003; Stone et al., 1995; West et al., 2005). This oxygen-sensing ability is essential for astrocytic VEGF-A expression and hence for retinal angiogenesis (Rattner et al., 2019; Stone et al., 1995). However, beyond this gene regulatory role, it was not known whether oxygen has a direct impact on astrocyte development. Previous studies addressing this question found only minor effects on astrocyte morphology or maturation when rodents were exposed to high O_2_ (Duan et al., 2017; Zhang et al., 1999). Here, we show that mitotic activity of neonatal astrocytes is bidirectionally regulated by oxygen: hyperoxia from P0 to P4 suppresses proliferation whereas direct hypoxia promotes proliferation. Our data are consistent with a model in which astrocytes sense local tissue oxygen levels using the HIF2α pathway, which drives proliferation in a manner proportional to the amount of HIF signaling. Such a mechanism would serve retinal metabolic needs by ensuring the addition of more VEGF-A^+^ angiogenic astrocytes when the tissue is hypoxic, and by suppressing this process once vessels have arrived to relieve local hypoxia. Manipulations of environmental oxygen appear to disrupt this homeostatic mechanism, such that decrements from baseline oxygenation are sufficient to drive HIF activity and excessive astrocyte proliferation.

The pro-proliferative effect of hypoxia is likely limited to a brief period of astrocyte development, as astrocyte proliferation has not been reported in the room-air phase of the standard (P7-P12) OIR paradigm. By P7, retinal astrocytes are substantially more mature: They have completed migration, ceased proliferating, and assumed a mature GFAP-expressing molecular profile (Chan-Ling et al., 2009; O'Sullivan et al., 2017; West et al., 2005). It is plausible that mature astrocytes may respond differently to metabolic stresses. Furthermore, most retinal astrocytes reside in close contact with vasculature by P7 – another key change that could contribute to the difference between the two oxygen regimes.

### Role of HIF2α in astrocytes: HIF signaling governs VEGF-A expression and astrocyte proliferation

Previous studies have yielded conflicting results regarding the function of HIF2α within astrocytes. Using astrocyte-specific *GFAP-Cre* mice, Duan and colleagues showed that vasculature was completely absent in mutants (Duan et al., 2014), likely due to the absence of VEGF-A expression (Rattner et al., 2019). Conversely, another study using a different *GFAP-Cre* mouse failed to find any effect of astrocyte Hif2α deletion (Weidemann et al., 2010). One possible explanation for this discrepancy is that the efficacy of the *GFAP-Cre-*mediated gene deletion might be variable. Here, we monitored HIF pathway activity in AC-Hif2α-KO mice by staining for the canonical HIF target VEGF-A. Using this approach, we validated that the *GFAP-Cre* line used in the Duan studies (Zhuo et al., 2001) does abrogate HIF function. This was shown by absence of VEGF-A expression from a large fraction of AC-Hif2α-KO astrocytes at P2, and the complete absence of astrocytic VEGF-A in at least a subset of mutants at P8-P10. In these ‘VEGF-low’ retinas with confirmed ablation of HIF signaling, vasculature was completely absent, as previously reported by Duan et al. (2014). Thus, our results clarify the essential role of astrocytic HIF2α for retinal angiogenesis, resolving a key discrepancy in the literature.

Our VEGF-low mutants exhibited another phenotype reported by Duan et al. (2014) – a major decrease in astrocyte abundance. Duan et al. (2014) argued that this phenotype was caused by decreased production of astrocytes from their neuroepithelial progenitors; they did not detect any change in astrocyte proliferation. Here, however, using VEGF-A staining to identify astrocytes deficient for HIF signaling, we demonstrate a cell-autonomous proliferation defect in mutant astrocytes. This phenotype was evident both in normoxia and when a strong pro-mitotic stimulus, i.e. relative hypoxia, was delivered via the NOIR protocol. Conversely, when the HIF pathway becomes hyperactivated in astrocytes, astrocyte numbers are increased (Duan and Fong, 2019). Together, these observations support our model whereby astrocyte proliferation rates are proportional to the amount of HIF signaling. The mechanism by which HIF2α promotes proliferation will be an interesting topic for future studies.

By P8-P10, we detected a ‘VEGF-high’ subset of AC-Hif2α-KO animals with normal astrocyte numbers and normal pattern of astrocytic VEGF-A expression. Nevertheless, a majority of these VEGF-high mutants exhibited delays in retinal angiogenesis (Fig. 8), strongly suggesting that astrocytic HIF function was not, in fact, entirely normal. We suggest that the VEGF-high phenotype can be explained by animal-to-animal variability in *GFAP-Cre* recombination efficiency. Cre^–^ astrocytes retaining HIF function are more proliferative than astrocytes with successful HIF2α knockout (Fig. 7D). Therefore, if the Cre^–^ population is large enough, it is conceivable that this competitive advantage could allow wild-type (i.e. VEGF-A^+^) cells to colonize the entire retina. Under these circumstances, it is likely that retinal angiogenesis would be delayed until the number of VEGF-A^+^ astrocytes became sufficient to drive vascular development. These results point to a potential source of confusion for past studies of HIF and VEGF-A in astrocytes (Duan et al., 2014; Weidemann et al., 2010), and highlight the need to validate knockout efficacy on a cell-by-cell basis when genes affecting proliferation are targeted.

### Neonatal hyperoxia alters the trajectory of retinal vascular development

Vascular development was disrupted in two distinct ways by the high-O_2_ and the low-O_2_ phases of the NOIR protocol. In the high-O_2_ phase, initiation of angiogenesis was blocked, consistent with previous reports (Claxton and Fruttiger, 2003; Morita et al., 2016; West et al., 2005). Only after return to normoxia did retinal vessels sprout from the ONH. Meanwhile, the embryonic hyaloid vascular system, which normally regresses in conjunction with elaboration of the intrinsic retinal vessels, persists and in some cases colonizes the neural retina (Fig. 2E). Surprisingly, high-O_2_ exposure had substantially different effects on vasculature when treatment was started during angiogenesis (Fig. S1) rather than before it (Fig. 1). This observation suggests that initiation of retinal angiogenesis is a distinct process from peripheral extension of intraretinal vessels, and that each process responds differently to oxygen.

During the low-O_2_ phase of the NOIR protocol, when retinal angiogenesis does eventually begin it does not progress normally (Figs 2 and 5). These findings differ from a recent study that exposed CD-1 mice to P0-P4 hyperoxia, in which the effects on retinal vasculature were reported to be mild and transient (Morita et al., 2016). The reason for the discrepancy between the two studies is unclear, as key aspects of study design – including the O_2_ percentage used – were similar. By contrast, our results are consistent with pathologies seen in young adult mice exposed to high oxygen from P0 to P7 (McMenamin et al., 2016). Together, these findings suggest that initiation of hyperoxia earlier than the traditional OIR model (Smith et al., 1994) can produce a different vascular phenotype, with the vitreoretinopathy and tractional detachments potentially mirroring some aspects of ROP (Foos, 1987) that are not well modeled by OIR (Gariano, 2010). Therefore, the NOIR protocol may serve as a useful complement to traditional OIR.

Our results from the NOIR model show a strong correlation between the magnitude of astrocyte overproduction and the severity of vascular delay/disarray. Because astrocyte phenotypes precede vessel growth and occur in peripheral retina prior to the arrival of vessels, we favor a model in which relative hypoxia affects astrocytes, and astrocytes in turn contribute to pathologic retinal vascularization. This would be consistent with other manipulations of astrocyte abundance during development, each of which demonstrate that vascular development is highly sensitive to the number of astrocytes within the angiogenic template (Duan and Fong, 2019; Fruttiger et al., 1996; O'Sullivan et al., 2017; Puñal et al., 2019; Tao and Zhang, 2016). However, our experiments cannot exclude the possibility that additional factors, including astrocyte responsiveness to vascular signals (Mi et al., 2001; Sakimoto et al., 2012; Selvam et al., 2018), may also contribute to the correlation between astrocytic and vascular phenotypes. Further work will be required to clarify the mechanisms by which astrocytes and endothelial cells interact during development and disease.

### Implications for ROP

Defining features of ROP, such as delayed peripheral vascularization, astrocyte hyperproliferation and long-lasting retinopathy, have been challenging to model in the mouse and are absent in the classic P7-P12 mouse OIR model (Gariano, 2010). The NOIR protocol we employed here is better able to model these disease features. Therefore, although traditional mouse OIR remains an important model, we expect that the NOIR model will become a useful complementary approach. The NOIR model should be particularly helpful in studying ROP variants that arise at earlier stages of retinal development than those modeled by traditional OIR. As improvements in neonatology improve survival rates for the most premature infants, models of these early retinopathies may become increasingly relevant to human disease.

Current treatment strategies for ROP do not target the early Phase I of the disease when angiogenesis becomes delayed. Instead they target Phase II, aiming to reduce neovascularization either by ablating ischemic peripheral retina or by blocking VEGF signaling. Our results suggest that inhibiting hypoxia-induced astrocyte overproduction could be a promising therapeutic avenue for preventing ROP vessel pathology, rather than mitigating damage as in current treatments. In our experiments, we were unable to assess whether suppressing astrocyte proliferation improved vessel phenotypes, because AC-Hif2α-KO mutants lacked all intrinsic retinal vasculature (Fig. 8). To test whether astrocyte proliferation is a promising therapeutic target, it will be necessary to identify additional and more selective molecular determinants of hypoxia-induced proliferation.

Our study sets the stage for identifying such factors and for testing their role in vascular pathology. With the NOIR protocol, we provide an experimental model for addressing the mechanisms of hypoxia-induced astrocyte proliferation and their consequences for retinal pathology. This approach should facilitate identification of molecular pathways that suppress astrocyte proliferation without interfering with angiogenesis, and could thus serve as targets for future therapeutics.

## MATERIALS AND METHODS

### Mice

All experiments were conducted with the approval of the Duke University IACUC. Timed pregnant CD-1 mice were purchased from Charles River (Wilmington, MA, USA) and C57BL/6J mice were purchased from The Jackson Laboratory (Jax stock 000664). *GFAP-Cre* mice, with the human *GFAP* promoter driving expression of Cre recombinase (Zhuo et al., 2001), were acquired from The Jackson Laboratory (Jax stock 004600) and backcrossed onto the C57BL/6J background for at least eight generations before use in these studies. HIF2α-flox (*Epas1^tm1Mcs^*) mice (Gruber et al., 2007) on a mixed 129X1-SvJ-C57Bl6/J background were also acquired from The Jackson Laboratory (Jax stock 008407). Animals of both sexes were used for all experiments.

Experimental design for NOIR and hypoxia experiments was as follows. For experiments with CD-1 mice, a cohort of two to four timed-pregnant females were monitored at ∼8 h intervals to identify the time of birth. Litters that were not delivered within 8 h of the rest of the cohort were removed from the experiment. For the remaining litters, pups were randomly assorted and cross-fostered amongst the dams, in order to control for litter-specific effects and minor variation in birth timing. Each cage, containing a dam and the mixed pup population, was assigned either to the control (normoxic) or experimental (high- or low-O_2_) condition. Within each experiment, the number of pups per cage was precisely matched between control and experimental groups. Across all experiments, the number of pups per cage was between eight and 12, all of which were used for phenotypic analysis. Mice from a single cage were collected at multiple time points. Four independent cohorts of mice were used for the high-O_2_ experiments and two independent cohorts were used for the low-O_2_ experiments.

For experiments with C57BL/6J and *GFAP-Cre*; *Hif2*α*-flox* mouse strains, it was not possible to use the cross-fostering strategy because we did not have access to multiple synchronously born litters. Instead, individual dams and their litters were exposed either to normoxia or high-O_2_ conditions. Litter sizes were six to nine pups. All wild-type and mutant animals were assessed phenotypically; *flox/+* heterozygotes were typically not analyzed although they were present in the cage during the experiment.

### Environmental oxygen manipulation

Cages with litters of mice and their mothers were placed inside an environmental chamber (A15274P, BioSpherix) to regulate oxygen concentrations. Medical O_2_ or N_2_ was mixed with room air by a regulator and O_2_ concentration calibrated and monitored with an O_2_ sensor (ProOx Model 360, BioSpherix); 75% O_2_ was used for hyperoxia and 10% O_2_ for hypoxia.

### Immunohistochemistry

Mice were anesthetized with ice or isoflurane and rapidly decapitated; eyes were removed and immersion fixed in 4% paraformaldehyde for 1.5 h at 4°C before being stored in PBS at 4°C. For flat-mounts, retinas were dissected free from the fixed eyes and blocked at room temperature for 1 h in PBS with 0.03% Triton X-100 (Sigma-Aldrich) and 3% normal donkey serum (Jackson ImmunoResearch). Retinas were then stained with primary antibody for 5-7 days at 4°C, washed three times with PBS, and then stained with donkey secondary antibodies (Jackson ImmunoResearch, ‘ML’ class cross-adsorbed antibodies; see Table S1) at a standard dilution of 1:1000. Following immunostaining, four relieving cuts were made in the retinas and they were flat-mounted on cellulose membranes (Millipore HABG01300) on glass slides and coverslipped with Fluoromount-G (Southern Biotech).

For cryosections, fixed whole eyes were sunk in 30% sucrose in PBS overnight and then 20 µm sections were cut using a cryostat. Sections were hydrated for 10 min with PBS and blocked for 30 min in PBS with 0.03% Triton X-100 (Sigma-Aldrich) and 3% normal donkey serum (Jackson ImmunoResearch). Sections were incubated in primary antibodies overnight, washed three times with PBS, stained with secondary antibodies for 2 h, and washed twice with PBS before mounting with Fluoromount-G.

*Griffonia simplicifolia* Isolectin B4 (IB4; 1:100, Life Technologies), conjugated to Alexa 488 (I21411) or biotin (I21414), was included with primary antibodies to stain blood vessels. Primary antibodies used were as follows: goat anti-GFAP (1:1000, Abcam ab53554; RRID:AB_880202); rat anti-Ki67 (1:3000, eBioscience 14-5698-80; RRID:AB_10853185); mouse anti-neurofilament (1:1000, EMD Millipore MAB1621; RRID:AB_94294); rabbit anti-Pax2 (1:200, Covance PRB-276P; RRID:AB_291611); rat anti-PDGFRα (1:1000, BD Biosciences 558774; RRID:AB_397117); rabbit anti-Sox9 (1:4000, Millipore AB5535; RRID:AB_2239761); and goat anti-VEGF-A (1:500, R&D Systems, AF-493-SP; RRID:AB_354506). All antibodies are well validated for the purposes employed here (see RRID profiles for further information). The antibodies to Pax2, Sox9, PDGFRα and GFAP have been validated as selective markers of retinal astrocytes (Puñal et al., 2019). The antibody to VEGF-A recognizes a band of expected size in western blots from neural tissue (Yu et al., 2014); detects VEGF-A immunohistochemically in mouse tissues in a manner that depends on HIF pathway activity (Blouw et al., 2007); and stains developing mouse retina in a pattern that recapitulates the expression pattern of *Vegfa* mRNA – including regulation by tissue hypoxia (Morita et al., 2017).

### Microscopy and image analysis

Retinas were imaged on a Nikon A1R confocal laser scanning microscope with 4× air, 20× air or 60× oil immersion objective lenses. A resonant scanner and motorized stage were used to acquire *z*-stacks or whole-retina tile scan images. For whole-retina images, tiles were stitched in Nikon NIS-Elements software (version 4.5). As a result of the automated software stitching process, minor stitching artifacts may be visible in some images. En-face images of vasculature depict *z*-projections of confocal stacks. The projected slices encompassed the innermost vascular plexus to the extent possible, although in some images some deeper vasculature is also visible. For all images, *z*-slices of interest were maximum-projected and denoised by median filtering using Fiji (radius 0.5-2.0 pixels). Brightness and contrast were then adjusted in Fiji and/or Adobe Photoshop. In some cases, minor nonlinear brightness-contrast adjustments were made. However, in all cases, the final adjusted image provides a faithful representation of the structures visible in the original image/*z*-stack. For multi-panel figures depicting different conditions of the same experiment (e.g. different genotypes or treatment conditions), image adjustment parameters were applied in a consistent manner to each image from each condition.

Cell counting and image analysis was performed in Fiji (Schindelin et al., 2012). Sox9 and Sox9/Ki67 double-positive astrocytes were counted manually, blinded to treatment group, in 60× images obtained from central, mid-peripheral and peripheral eccentricities. Total astrocyte numbers were estimated by multiplying retina area by average weighted astrocyte density; three or four fields of view from central, mid-peripheral and peripheral retina were counted, mean density for each eccentricity in each retina calculated, and overall average density calculated by weighting central, middle and peripheral eccentricities with factors of 0.11, 0.33 and 0.56, respectively, based on a circular approximation of the retina divided into three zones by concentric rings with radii of 1×, 2× and 3× (O'Sullivan et al., 2017). For hypoxia experiments, we found that rearing mice in 10% O_2_ inhibited migration of astrocytes into peripheral retina. Therefore, to avoid confounding the proliferation analysis, we limited our quantification of Ki67^+^ astrocytes to the central region that astrocytes did colonize. Further, to control for the possibility that astrocytes outside the vascular wavefront might experience hypoxia regardless of circulating oxygen levels, images were taken from the vascularized region or at the vascular wavefront. In the latter case, images were framed with the wavefront in the center of the region of interest (size=106 µm^2^), such that all astrocytes analyzed for this experiment were either within the vascular region or at most ∼53 µm away from vessels. As such, the vast majority of quantified astrocytes were expected to have access to oxygen circulating within the vasculature.

### Astrocyte and blood vessel coverage

Low-magnification tile-scan images of whole retinas were used to assess retinal coverage by astrocytes and blood vessels. The retinal perimeter was manually traced in ImageJ, and within that perimeter a second curve encompassing the furthest peripheral Sox9- or IB4-stained area was drawn and its area measured to yield astrocyte and blood vessel area, respectively (Schindelin et al., 2012).

### Analysis of HIF2α mutant astrocytes: VEGF-A expression and proliferation

To assess HIF pathway activation on a cell-by-cell basis, retinal whole-mounts were stained with VEGF-A, Sox9 and Ki67 antibodies. Sox9^+^ astrocytes were classified as VEGF-A^+^ or VEGF-A^–^ in 60× confocal image stacks. For proliferation analysis at P2, the fraction of Ki67^+^ astrocytes in each category was quantified from three *GFAP-Cre;* Hif2α-flox mutants and three wild-type littermates. Data were plotted as the 95% confidence interval of the sample proportion (2151 wild-type astrocytes and 1356 mutant astrocytes from 3 mice of each genotype). To assign P8-P10 *GFAP-Cre;* Hif2α-flox mutants as either ‘VEGF-high’ or ‘VEGF-low’, we assessed 20× and 60× image stacks and examined tissue by eye. With only one exception, all mutants examined could be easily assigned to one of these categories because the vast majority of the astrocyte population was either VEGF-A^+^ or VEGF-A^–^ (*n*=17 mutants from 5 separate litters). The one retina that contained a large proportion of both VEGF-A^+^ and VEGF-A^–^ astrocytes was excluded from analysis.

### Data analysis

Statistical analyses were performed in GraphPad Prism 8 (GraphPad Software) or JMP Pro 13 (SAS Institute). Tests for statistical significance included two-tailed unpaired *t*-tests; one-way ANOVA with post-hoc Tukey's test; and two-way ANOVA with post-hoc Holm–Sidak test. Post-hoc test *P*-values were corrected for multiple comparisons. Summary statistics are reported as mean±s.d., and error bars display s.d. (except where noted). Unless otherwise noted, data points on graphs represent measurements from one eye of one individual animal.

## Supplementary Material

Supplementary information

Reviewer comments
