# Supplementary material for: Environmental oxygen regulates astrocyte proliferation to guide angiogenesis during retinal development
Source: Development. 2021 May 7;148(9):dev199418. doi: 10.1242/dev.199418 (PMC8126409; doi:10.1242/dev.199418)
Supplement: Supplementary information [file develop-148-199418-s1.pdf]

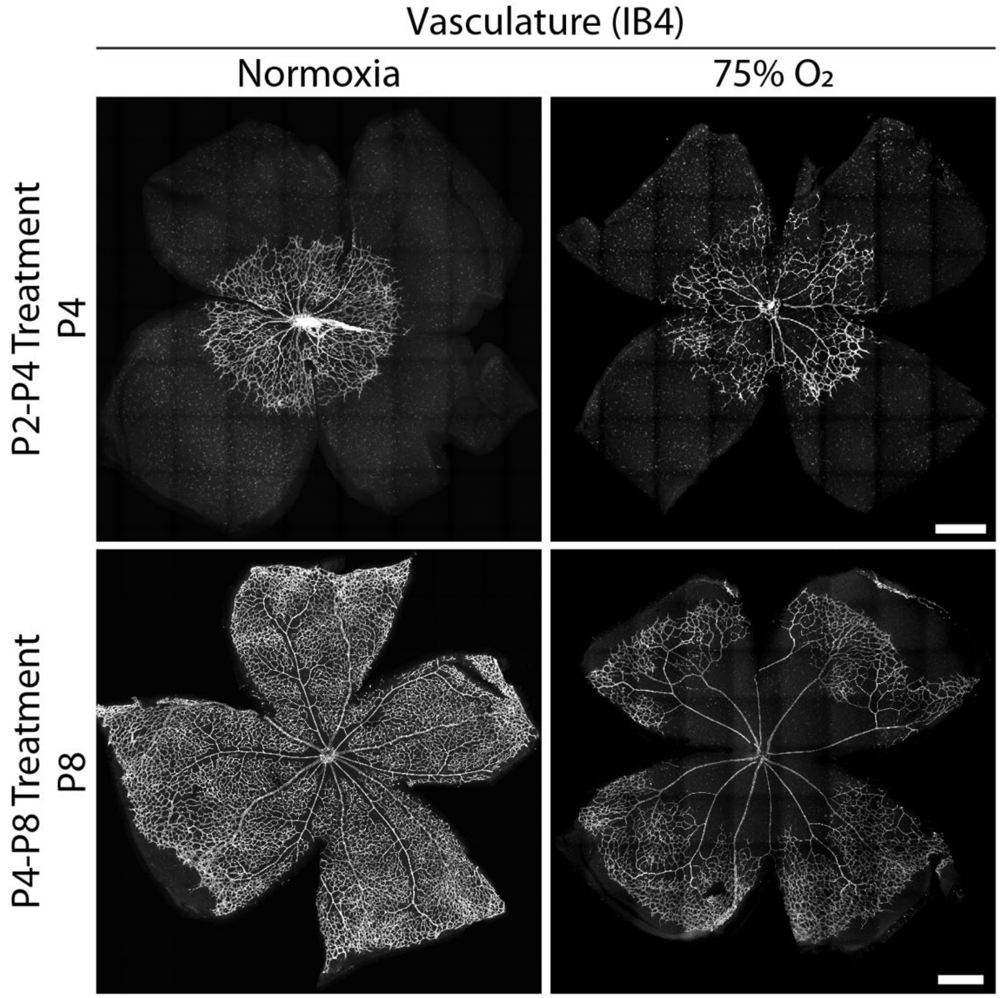


**Figure S1. Effects of starting hyperoxia at P2 or P4 on vascular development**.

Representative whole-mount images of retinal vasculature from animals reared in 75% $O_2$ from P2-P4 (top) or P4-P8 (bottom). Animals were sacrificed at the end of the high-$O_2$ treatment (i.e. P4 or P8, respectively). Progression of vascular wavefront is similar to normoxic littermate controls in both treatment paradigms. However, central vaso-obliteration is observed in treated animals, similar to standard P7-P12 OIR paradigm. Scale bars, 500 $\mu$m.

Development • Supplementary information

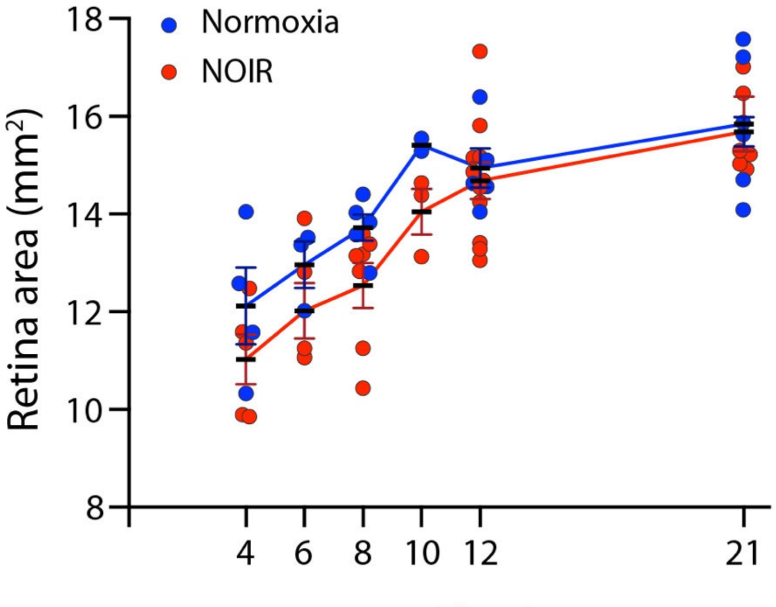


**<u>Figure S2. Overall growth of the retina is not disturbed by return to room air in the NOIR protocol</u>.**
To learn whether the NOIR protocol causes non-specific eye growth abnormalities or developmental delays, retinal area was measured across development in NOIR-exposed animals (red) and normoxic littermate controls (blue). Each data point represents one retina measurement from one animal. Statistics, two-way ANOVA with post-hoc Sidak test. The size of the retina was not significantly different at any age examined (see below for P values). We did note a subtle rightward shift in the growth curve; accordingly, a two-way ANOVA showed a main effect of oxygen treatment ($F(5, 51) = 20.72$; $p = 1 \times 10^{-7}$). The curve shift was already present at P4, indicating that this effect was due to the high-$O_2$ phase of the NOIR protocol. Subsequently, between P4 and P21, the retinal growth rate (i.e. slope of the size curves) was similar in normoxia and NOIR animals. Thus, retinal growth is normal during the relative hypoxia phase of the NOIR protocol when astrocyte and vascular phenotypes first emerge. P-values for post-hoc Sidak tests: P4, p = 0.6233; P6, p = 0.8260; P8, p = 0.3785; P10, p = 0.7117; P12, p = 0.9987; P12, p = 0.9999. Error bars, S.E.M.

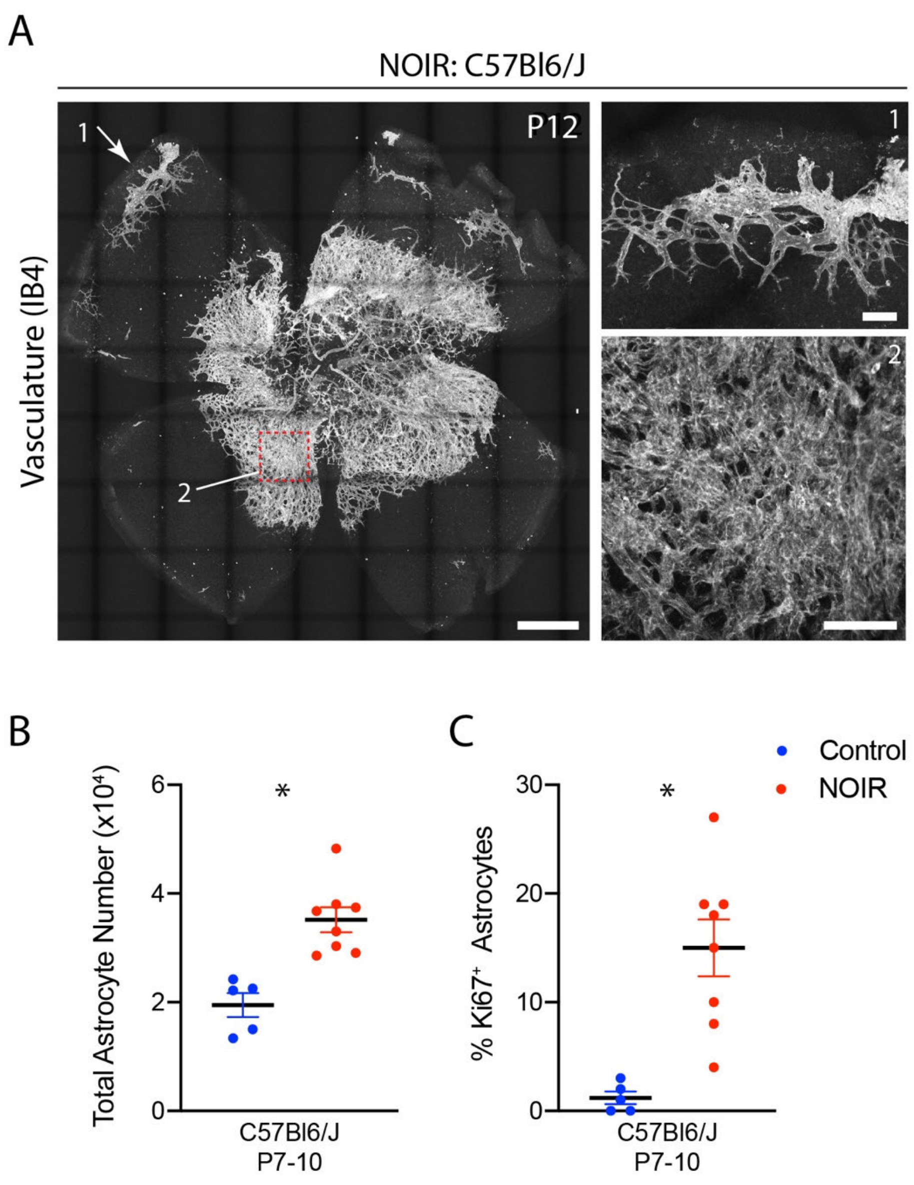


**<u>Figure S3</u>. <u>NOIR phenotype in C57Bl/6J mice is similar to CD-1</u>.**

**A**) Representative images illustrating retinal vascular phenotypes in NOIR-exposed C57Bl/6J (C57) animals at P12. As in CD-1 (Fig. 2), the P12 C57 NOIR retina exhibits an extensive peripheral avascular zone, absence of large radial vessels, and hyperdense and irregular vascularity centrally. Boxed region is shown at high magnification in panel 2, illustrating capillary disorganization and lawn-like features, similar to the CD-1 phenotype (Fig. 2D). Arrow and panel 1 illustrate defects in peripheral retina – vitreous-derived vasculature initiating angiogenesis back towards central retina. This phenotype was also present in CD-1 (Fig. 2E). Scale bars, 500 $\mu$m (whole-retina view, left); 100 $\mu$m (panels 1 and 2, right).

**B,C**) Quantification of astrocyte number (B) and proliferation (C) in C57 NOIR animals following return to room air. Similar to CD-1 mice (Fig. 3), NOIR exposure increases both the fraction of proliferating astrocytes (C) and total astrocyte number (B) relative to normoxic C57 controls. Each data point represents a different animal. Statistics, two-tailed t tests. *(B) p = 0.0008. *(C) p = 0018. Error bars, mean ± s.e.m.

Development • Supplementary information

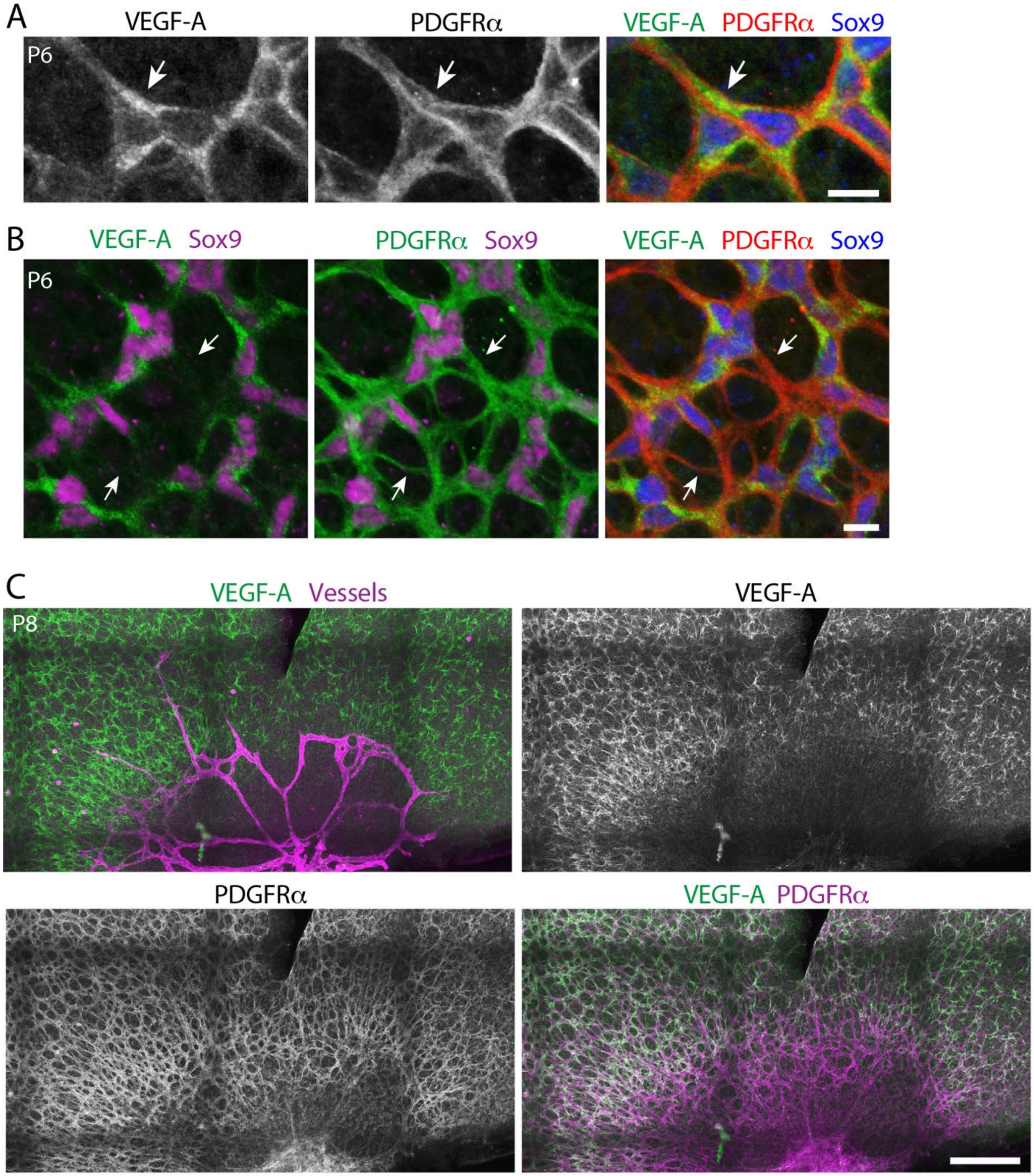


**Figure S4. Characterization of VEGF-A immunoreactivity in retinal astrocytes**.

**A,B**) En-face views of retinal astrocytes in wild-type normoxic mice, labeled for VEGF-A and two astrocyte markers (Sox9, PDGFRα). Confocal z-stacks were acquired from P6 retinas in the avascular periphery; panels depict z-projections encompassing the depth of the RNFL. Virtually all astrocytes express VEGF-A. High-magnification view (A) shows that VEGF-A immunoreactivity (green) localizes almost exclusively to intracellular organelles located within the bounds of the cell-surface PDGFRα marker (red). Little if any VEGF-A signal was detected on or outside the bounds of the PDGFRα$^+$ plasma membrane. Lower-power images (B)

Development • Supplementary information

show that VEGF-A immunoreactivity is excluded from PDGFRα$^{+}$ astrocyte arbors. Morphology and perinuclear location of intracellular VEGF-A$^{+}$ structures (A,B) is consistent with staining of secretory pathway organelles (i.e. endoplasmic reticulum and/or Golgi apparatus). Together, these observations suggest that the antibody to VEGF-A is capable of detecting high protein concentrations within the cells that are secreting it, but is not sensitive enough to reveal lower concentrations of extracellular secreted protein.

**C**) Representative example of VEGF-A expression in NOIR retina. Astrocytes were labeled using PDGFRα. As in wild-type (Fig. 7A), VEGF-A is highly expressed by astrocytes in avascular retinal regions, whereas astrocytes in vascularized regions express little VEGF-A. Scale bars, 10 $\mu$m (A,B); 200 $\mu$m (C).

Development • Supplementary information

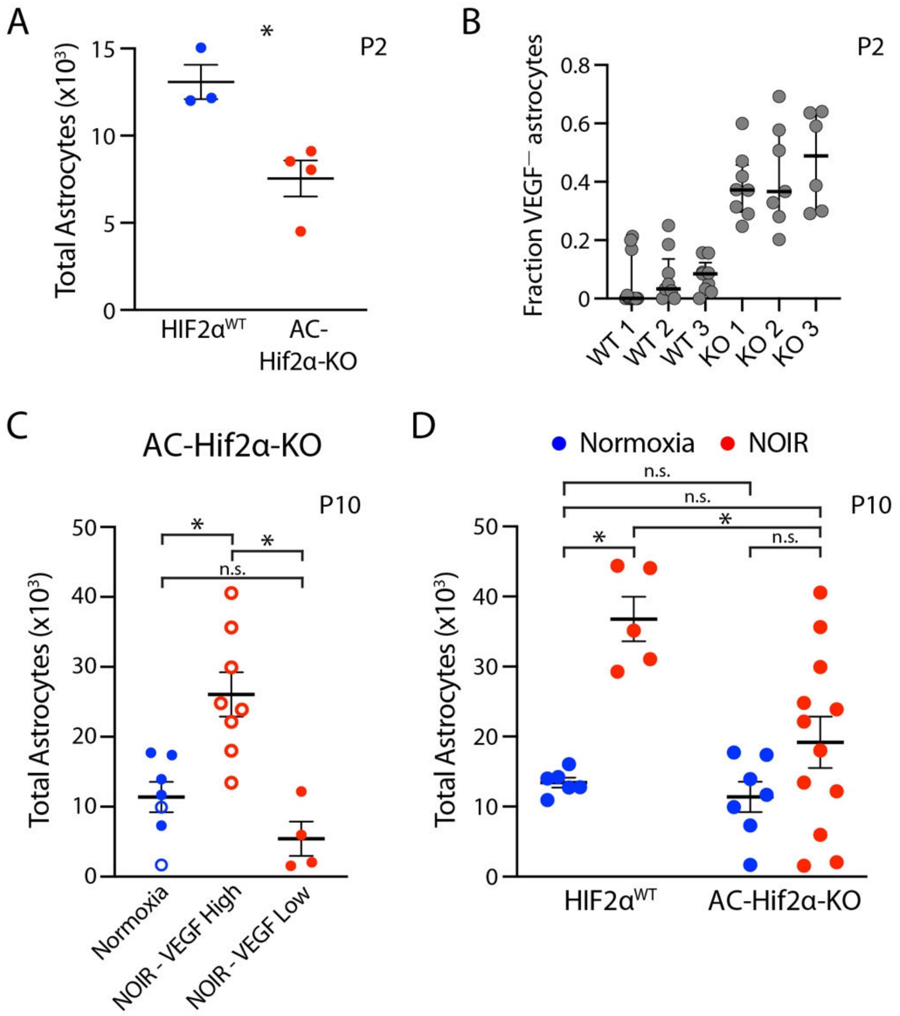


**Figure S5. Characterization of AC-Hif2α-KO mutants**. **A**) At P2, AC-Hif2α-KO mutants have fewer astrocytes than littermate controls. Statistics: Two-tailed t-test, *p = 0.0134. **B**) Evaluation of HIF signaling status in wild-type (WT) and AC-Hif2α-KO (KO) astrocytes at P2, tested via VEGF-A staining. Data from three retinas of each genotype (each retina from a different animal). Tissue was stained as in Fig. 7C, and the fraction of VEGF-A$^-$ Sox9$^+$ cells was quantified from 106 $\mu m^2$ fields of view. Points represent individual measurements made at multiple different eccentricities. Elimination of HIF signaling is highly variable in P2 AC-Hif2α-KO mutants, even within a single retina. **C**) At P10, the VEGF-high and VEGF-low classes of AC-Hif2α-KO mutants have significantly different astrocyte production responses in the NOIR paradigm. This finding provides further evidence that the two classes of mutants are fundamentally different and should be considered different experimental groups. Normoxia group includes both VEGF-low (open circles) and VEGF-high (closed circles) KO mice. One-way ANOVA, F (2,16) = 13.5, p = 0.0004. Asterisks denote significant differences by Tukey's post-hoc test. VEGF-high vs. VEGF-low, p = 0.0007. Normoxia vs. VEGF-high, p = 0.0032. Normoxia vs. VEGF-low, p = 0.4079. **D**) Total astrocyte numbers at P10 in AC-Hif2α-KO mutants and littermate controls. Graph is the same as Fig. 9C, except that all AC-Hif2α-KO mutants have been plotted regardless of VEGF-A status. Even when no distinction is made between VEGF-high and VEGF-low mutants, there is still a significant effect of genotype on astrocyte number (Two-way ANOVA, main effect of genotype F(1,26) = 7.780 , p = 0.0098 ; main effect of oxygen F(1,26) = 19.49 , p = 0.0002 ; interaction, F(1,26) = 4.846 , p = 0.0368). Furthermore, a post-hoc test (Holm-Sidak) shows that loss of Hif2α significantly blunts the effect of NOIR exposure even when VEGF-high animals are included in the analysis (control NOIR vs. mutant NOIR, p = 0.0051). Consistent with this finding, we did not detect a significant astrocyte number difference between

Development • Supplementary information

normoxic and NOIR mutants (Holm-Sidak, $p = 0.2337$). Therefore, even though there is strong biological justification for excluding VEGF-high mutants from the experiment, failing to do so does not change our central conclusion that Hif2α is required for the astrocyte proliferative response in the NOIR paradigm. Other P values noted on graph: normoxia controls vs. normoxia mutants, $p = 0.6874$; normoxia controls vs. hyperoxia mutants, $p = 0.3947$. Error bars: A,C,D, mean ± s. e. m.; B, mean and interquartile range.

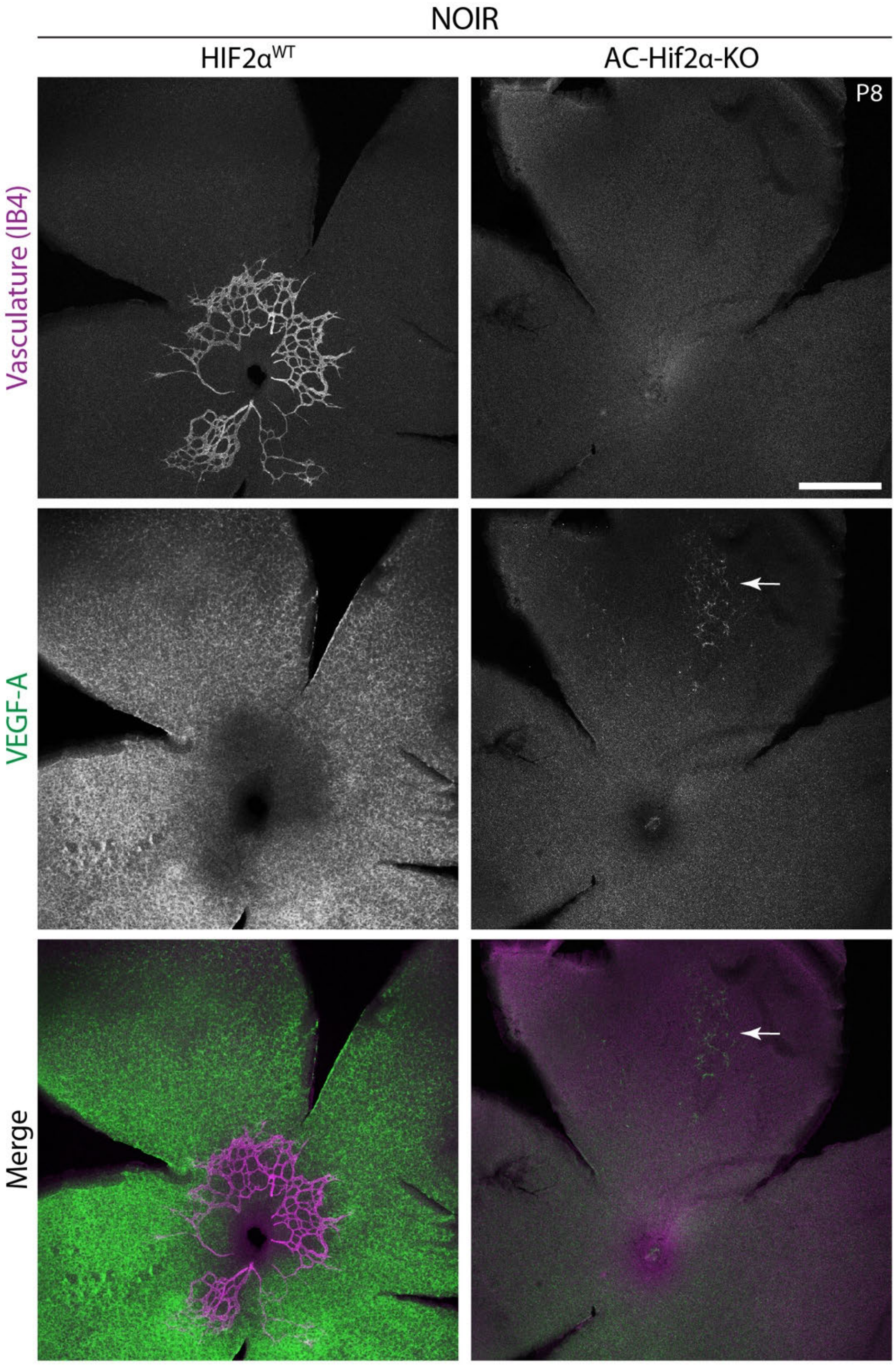


**Figure S6. Vasculature and VEGF-A expression in NOIR retinas**

Representative examples of Hif2α$^{WT}$ and AC-Hif2α-KO NOIR retinas four days after return to room air (P8), stained for vasculature and VEGF-A. In control WT retinas, VEGF-A becomes strongly upregulated in avascular retina, but mutants (VEGF-low category) fail to upregulate VEGF-A. Arrow indicates a small cluster of VEGF-A$^{+}$ astrocytes in mutant retina, likely due to incomplete *GFAP-Cre* activity. Vasculature is entirely absent from VEGF-low AC-Hif2α-KO mutants, regardless of whether they were NOIR exposed (this figure, top panels) or raised in normoxic conditions (Fig. 8). Scale bar, 500 $\mu$m.

**Table S1. Secondary antibodies used in this study**

| Host | IgG Specificity | Fluorophore | Vendor | Catalog # |
|---|---|---|---|---|
| Donkey | anti-goat | Alexa 488 | Jackson ImmunoResearch | 705-545-147 |
| Donkey | anti-goat | Cy3 | Jackson ImmunoResearch | 705-165-147 |
| Donkey | anti-goat | Alexa 647 | Jackson ImmunoResearch | 705-605-147 |
| Donkey | anti-mouse | Alexa 488 | Jackson ImmunoResearch | 706-605-148 |
| Donkey | anti-mouse | Cy3 | Jackson ImmunoResearch | 715-165-151 |
| Donkey | anti-mouse | Alexa 647 | Jackson ImmunoResearch | 715-605-151 |
| Donkey | anti-rabbit | Alexa 488 | Jackson ImmunoResearch | 711-545-152 |
| Donkey | anti-rabbit | Cy3 | Jackson ImmunoResearch | 711-165-152 |
| Donkey | anti-rabbit | Alexa 647 | Jackson ImmunoResearch | 706-605-148 |
| Donkey | anti-rat | Alexa 488 | Jackson ImmunoResearch | 712-545-153 |
| Donkey | anti-rat | Cy3 | Jackson ImmunoResearch | 712-165-150 |
| Donkey | anti-rat | Alexa 647 | Jackson ImmunoResearch | 712-605-153 |
