# Supplementary material for: Environmental oxygen regulates astrocyte proliferation to guide angiogenesis during retinal development
Source: Development. 2021 May 7;148(9):dev199418. doi: 10.1242/dev.199418 (PMC8126409; doi:10.1242/dev.199418)
Supplement: Reviewer comments [file develop-148-199418_review_history.pdf]

Environmental oxygen regulates astrocyte proliferation to guide angiogenesis during retinal development

Robin M. Perelli, Matthew L. O'Sullivan, Samantha Zarnick and Jeremy N. Kay
DOI: 10.1242/dev.199418

Editor: Stephen Wilson

Review timeline
Transfer from Review Commons: 8 January 2021
Editorial decision: 23 February 2021
First revision received: 26 March 2021
Accepted: 7 April 2021

Submission to Review Commons

Reviewer 1

Evidence, reproducibility and clarity

In this study Perelli et al. demonstrated that exposure of mouse pups to hyperoxia from P0-P4, followed by returning to normoxia leads to overproliferation of retinal astrocytes, which appears to impact on retinal vascularization. The authors also showed that in astrocyte specific HIF2a KO mice the retinal astrocytes are present in reduced numbers and do not over proliferate in the P0-P4 hyperoxia model. These are interesting findings, but I have a few concerns:

Major
1. It is not clear how "penetrant" the P0-P4 model is. Do all the hyperoxia treated mice display the abnormal astrocyte/vessel network shown in Fig. 2 and Fig. 4 or is there a range of phenotypes? And furthermore, have all the animals in a given litter been included in the quantitations.

2.Following up on comment 1, quantitations in Fig. 6 appear to be based on 6 retinas (and I'm guessing 3 mice?). Again, how were these animals chosen from the litters of 8-10 pups mentioned in materials and methods. Furthermore, the authors should state more clearly how many different animals were analysed.

3.At which location in relation to the developing vasculature was astrocyte proliferation measured in the 10% hypoxia model (Fig. 6). The authors mention that they avoided the peripheral retina (due to lack of astrocytes there), but were all the measured astrocytes in "contact" with vessels? Presumably, astrocytes peripheral to vessels experience hypoxia irrespective of the oxygen in the breathing air, whereas astrocytes associated with vessels may indeed experience different oxygen levels depending on the oxygen availability in the blood. Have the authors controlled for this?

4.I'm intrigued by the immunostain for VEGFA, which is notoriously difficult because VEGFA protein is present at very low abundance. Fig. 7 is not sufficient proof that this stain is specific. Since KO controls are not feasible in this instance, the authors should demonstrate that the staining changes in hyperoxia and hypoxia exposed animals as predicted.

5. Following up on point 4, it is also possible that astrocytes bind VEGFA that has been produced by other cells, which means that there is not necessarily a correlation between VEGFA IHC positive astrocytes and hypoxic astrocytes.

© 2021. Published by The Company of Biologists under the terms of the Creative Commons Attribution License (https://creativecommons.org/licenses/by/4.0/).

6. What is the proportion of VEGFA negative astrocytes in the AC-Hif2a KOs (in comparison to all astrocytes)?

7. The image in Fig. 7B is not convincing at all. The background on the left side of the VEGF- A panel is clearly much darker, which also the location where all the VEGFA negative astrocytes happen to be. Also, at what location (in relation to the vessels) where the measurements shown in Fig. 7 C taken?

8.I am intrigued by the VEGFA stain in the "VEGF-low" AC-Hif2a KOs (Fig. 7 D). If there are no vessels, we can assume the retina to be fairly hypoxic and RGCs ought to express VEGFA strongly, why is this not picked up by the VEGFA stain?

9. The authors have shown profound GFAP upregulation in Muller cells (Fig. 5). In the AC-Hif2a KO background this should lead to VEGFA deletion also in Muller cells. Have the authors considered the confounding effects that might cause?

10. What are the outcomes for retinal vascularisation in the hyperoxia exposed AC-Hif2a KO mice?

Minor

11. What is the p value for the linear regression model of Fig4C?

12. The terms of hyperoxia and hypoxia can be confusing to non-specialist readers. So, whilst mice are exposed to hyperoxia, when they are returned to room air, the retinal astrocytes experience hypoxia.

Significance

The study is interesting and potentially useful, but at this stage not entirely convincing.

Reviewer 2

Evidence, reproducibility and clarity

The authors explored the effect of hyperoxygen exposure on glial and vascular development. They also analyzed the phenotype in astrocyte-specific Hif2 knockout mice. The conclusion is that astrocyte oxygen sensing through Hif2 regulates the number of astrocytes required for proper vascular development. Overall, the data are of good quality and convincing.

Significance

However, it's hard to identify any novel finding upon previous papers (Morita et al., Birth Defects Res B Dev Reprod Toxicol. 2016; Duan et al., Plos One 2014; Duan et al., Sci Rep 2017). The present study looks just a replication of previous ones listed above, although there are differences in mouse strains and in the severity of retinal degeneration.

Reviewer 3

Evidence, reproducibility and clarity

Summary:

The study by Perelli et al. explores the role of hypoxia-driven astrocyte proliferation in mediation of pathological angiogenesis during retinopathy of prematurity. The authors examine CD1 neonatal pups exposed to 75% oxygen from P0-P4, followed by analyses of the development of the astrocyte

© 2021. Published by The Company of Biologists under the terms of the Creative Commons Attribution License (https://creativecommons.org/licenses/by/4.0/).

network and the vasculature during the subsequent relative hypoxia period upon return to normoxia. The role of HIF2a in regulating VEGFA expression in astrocytes is examined using GFAP-Cre; Hif2a mice. Overall, this is an interesting study but very importantly, the genetic background of the mice and the establishment of vascular pathology have not been properly explained.

Major comments

1. The mouse genotype contributes very decisively to establishment at least of the vascular phenotype in retinopathy. The absolute majority of vascular analyses as a consequence of OIR is done using C57Bl/6. In some strains such as FVB or Balb/c, pathological angiogenesis is not seen in response to the P7-P12 regiment (Ritter et al., J Clin Invest. 2006 Dec 1; 116(12): 3266-3276). See for example O´Bryhim et al., Mol Vis. 2012; 18: 377- 389 with regard to impact of the mouse strain genetic background on avascularity in response to hyperoxia. Therefore, it is very important that the authors describe and are consistent, with regard to the strains they use in this study. Most of the analyses appears to have been done using CD1. Why was this strain chosen, and not C57? This should be motivated. How does vascular development {plus minus} hyperoxia compare between DC-1 and C57? The GFAP-Cre mice appear to be on FVB background at Jax (FVB-Tg(GFAP- cre)25Mes/J Stock No: 004600). Did the authors backcross these mice to CD-1 or use a mixed background? The Epas1floxed mice are in the SvJ background at Jax. The genetic backgrounds of the different strains are not described in the methods and the potential impact different genetics may have on the results is not considered. That is absolutely required. Also, I lack information on when and how tamoxifen was delivered and the extent of deletion. All pups should have received tamoxifen - this is essential information which is not given. Moreover, as it is known that the establishment of the eye pathology after high- oxygen is highly dependent on pup weight at the point of evaluation, this information must be given in order to clarify ascertain that the authors are studying a defect specific to retinal pathology and not due to a general developmental defect.

2. The authors make the point that developmental formation of the astrocytic network is a prerequisite for vascular development and that excess proliferation of astrocytes in hypoxia (relative or true) may drive the vascular pathologies seen in eye diseases such as retinopathy of prematurity. In Fig. 1, the authors show that no vessels are formed in the CD1 strain exposed to hyperoxia during P0-P4. When hyperoxia occurs during P2-P4, a large avascular area is formed at P8 similar to the "traditional" finding, meaning in C57 mice, I assume? Please explain. However, the effect of hypoxia-regulated VEGFA induction in response to the relative hypoxia after high O2 incubation, is in formation of pathological angiogenesis, so-called vascular tufts. Did vascular tufts become established in the CD1 strain after the "conventional" P7-12 exposure and after the P0-P4 exposure? If such tufts are not formed, conclusions on the development of the astrocytic network preceding the establishment of any hypoxia/VEGFA-dependent vascular pathologies cannot be made. The statement on p18 concerning "Starting hyperoxia at P0 has qualitatively different effects compared to later stage" with a reference to Louis Smith who uses C57, is not valid.

3. In Fig. 1B, dense IB4-positive patches are seen at P12. Are these tufts or sheets of ECs? This should be better described and shown at high magnification. It does not seem to resemble the peripheral sheets of endothelial cells highlighted in Fig. 2D.

4. On page 18, the authors state that 50% of the hyperoxia-treated mice had persistent pathology. I assume this is in the wildtype CD1 mice and unrelated to efficiency of floxing of Hif2a. This variable establishment of pathologies and which one may or may not be established, is not clear from the results except for the hemorrhaging. What is meant here with "persistent"? Please overall explain and present the vascular and astrocytic pathology and the penetrance in a clear manner.

Minor

5. In Fig 1 C, please make it clear which hyperoxia period the mice were exposed to.

6.Data in Fig 5 needs to be quantified.

7.The authors could try to immunostain for HIF2 to classify Cre+ mice as successfully deleted?

© 2021. Published by The Company of Biologists under the terms of the Creative Commons Attribution License (https://creativecommons.org/licenses/by/4.0/).

Significance

The role of VEGFA produced by astrocyte for vascular development is well established, however much less is known about a potential driving role of astrocytes in vascular pathologies such as in retinopathy. I therefore think this study would be of broad interest.

The presentation is good and referencing is adequate.

Key words: VEGF, OIR, vascular pathology, vascular tufts.

Author response to reviewers' comments

Response to reviewers - Perelli et al.
Here we provide a point-by-point response to all reviewer critiques. In italics we have quoted the reviewers fully, without editing, except that in the Reviewer 3 section we changed the order of certain related points to keep them together for clarity. These are denoted with ellipses. Please note that line numbers refer to the revised manuscript Word file with Track Changes turned to Simple Markup mode (or turned OFF).

Reviewer #1
(Evidence, reproducibility and clarity (Required)):

In this study Perelli et al. demonstrated that exposure of mouse pups to hyperoxia from P0-P4, followed by returning to normoxia leads to overproliferation of retinal astrocytes, which appears to impact on retinal vascularization. The authors also showed that in astrocyte specific HIF2a KO mice the retinal astrocytes are present in reduced numbers and do not over proliferate in the P0-P4 hyperoxia model. These are interesting findings, but I have a few concerns:

Major

1.1)*1.It is not clear how "penetrant" the P0-P4 model is. Do all the hyperoxia treated mice display the abnormal astrocyte/vessel network shown in Fig. 2 and Fig. 4 or is there a range of phenotypes?*

We regret that we were not initially clear on this critical point. While the original submission did provide some information about phenotype penetrance – e.g. variability of vessel delay (Fig. 1C), hemorrhage (Fig. 2A-B), and astrocyte number (Fig. 3B) – we agree that we did not give enough information about the penetrance of other key phenotypes in the P0-P4 NOIR model. To address this we have changed the following in our revised manuscript:

- Results (lines 255-268) and the legend to Fig. 2 have been revised to state how often we observed the various vascular phenotypes. We now make it clear that some phenotypes were consistent across animals, whereas for other phenotypes there was a range of severity.
- A new panel was added to Fig. 2 (middle panel, Fig. 2D) to better illustrate the range of vascular phenotypes.
- We now better describe the range of astrocyte patterning phenotypes – see Results (lines 333-340) and legend to Fig. 4.

1.2)*And furthermore, have all the animals in a given litter been included in the quantitations.*

Yes they have. The one exception is that we did not analyze Hif2α flox/+ heterozygotes. To clarify this point we made substantial additions to the Methods, describing in more detail our experimental design (lines 126-142). We also made additions to the Results section (lines 232-235) to summarize the approach. As noted in the new text:

- The CD-1 high-O2 experiments (i.e. NOIR experiments) comprise four separate independent cohorts of mice. Each cohort represents 2-4 age-matched litters that were cross-fostered so the litters would be mixed across control and experimental conditions. The hypoxia (low-O2)

© 2021. Published by The Company of Biologists under the terms of the Creative Commons Attribution License (https://creativecommons.org/licenses/by/4.0/).

experiments comprise two independent cohorts arranged in a similar fashion.

- The animals in any given cohort were collected at multiple timepoints. Each cage of 8-12 mice typically yielded 4 timepoints with 2-3 mice at each age.

1.3)*2.Following up on comment 1, quantitations in Fig. 6 appear to be based on 6 retinas (and I'm guessing 3 mice?). Again, how were these animals chosen from the litters of 8-10 pups mentioned in materials and methods. Furthermore, the authors should state more clearly how many different animals were analysed.*

It seems that there has been some confusion as to the meaning of individual data points on our graphs. We have now clarified this with an explicit statement in the Methods section (lines 227-8): "Unless otherwise noted, datapoints on graphs represent measurements from one eye of one individual animal." This is true for all graphs in the main figures, and most of the graphs in the supplemental material. Additionally, to specifically address the reviewer's question, we have also mentioned this important point in the legend to Fig. 6.

Thus, the quantifications in Figure 6 are actually based on 18 animals – 9 in the normoxia group and 9 in the hypoxia group – that were collected at two different timepoints (P2 and P4). As noted in Point 1.2 above, these 18 animals came from two independent cohorts of mice, all of which were collected and used for this experiment (although some mice from this cohort were stained with other markers besides Ki67 so they were not included on the graph or used in this particular quantification).

1.4)*3.At which location in relation to the developing vasculature was astrocyte proliferation measured in the 10% hypoxia model (Fig. 6). The authors mention that they avoided the peripheral retina (due to lack of astrocytes there), but were all the measured astrocytes in "contact" with vessels? Presumably, astrocytes peripheral to vessels experience hypoxia irrespective of the oxygen in the breathing air, whereas astrocytes associated with vessels may indeed experience different oxygen levels depending on the oxygen availability in the blood. Have the authors controlled for this?*

We agree with the reviewer that astrocytes located in the avascular retinal region might experience a different local oxygen environment than those inside the vascular wavefront. We did in fact control for this potential confound – we regret not explaining this more clearly in the initial submission.

All of the images analyzed for the experiment in Fig. 6, regardless of treatment group, were taken either inside the vascular region or right at the vascular wavefront. When the wavefront was included in the image, it was positioned in the middle of the field of view (106 μm^2), such that approximately half of the imaged territory was inside the vessels and half was outside. Therefore, the vast majority of astrocytes analyzed in this experiment were within the vascularized region. And even those located outside the wavefront were still very close to the vasculature – no more than ~ 53 μm away. Given this short distance most of these "outside" astrocytes were still probably within the range of oxygen diffusion.

Based on these details of our experimental design, we expect that circulating oxygen levels should be the major determinant of local oxygen environment for the astrocytes that were quantified in Fig. 6. We therefore see no reason to believe that differential access to vasculature could have confounded these results. We have now included the experimental details described above in the Methods section (lines 192- 201). We also note in the legend to Fig. 6 that all images were taken in central retina, within the vascular region.

1.5)*4.I'm intrigued by the immunostain for VEGFA, which is notoriously difficult because VEGFA protein is present at very low abundance. Fig. 7 is not sufficient proof that this stain is specific. Since KO controls are not feasible in this instance, the authors should demonstrate that the staining changes in hyperoxia and hypoxia exposed animals as predicted.*

The reviewer rightly notes how important it is to document specificity of the VEGF-A antibody (goat anti-mouse VEGF164; R&D Systems Cat # AF-493; RRID: AB_354506). We agree that doing this with a

© 2021. Published by The Company of Biologists under the terms of the Creative Commons Attribution License (https://creativecommons.org/licenses/by/4.0/).

Vegfa-flox mouse would not be feasible. So we have followed the reviewer's suggestion and added new evidence that VEGF-A immunoreactivity is regulated by oxygen levels, as would be expected based on previous studies demonstrating regulation of *Vegfa* mRNA by oxygen. To this end we have made three significant additions to the paper as follows.

First, we have performed the experiment requested by the reviewer. This experiment is described in the revised Results (lines 417-422) and new figures (Fig. 7B; Supplemental Fig. S4C).
Mice were exposed either to normoxia or 75% O_2 (high-O_2) starting at P0. VEGF-A immunoreactivity was then assessed at P2. Based on previous studies of *Vegfa* mRNA expression in the retina (e.g. Stone et al., 1995; PMID 7623107) we expected that high O_2 should suppress astrocytic VEGF-A. Indeed, that is exactly what we found: astrocyte VEGF-A immunoreactivity was severely diminished in the high-O_2 condition relative to normoxic controls (Fig. 7B).

We then asked what would happen to VEGF-A expression when O_2-exposed mice were returned to room air. We expected that this manipulation would restore astrocytic VEGF-A expression, because the decrement in oxygen levels should activate the HIF pathway leading to transcription of the *Vegfa* gene. Again, our findings with the VEGF-A antibody were precisely in line with expectations: In O_2-treated mice that were returned to room air, VEGF-A was once again expressed by astrocytes in avascular retina (see new figures Fig. S4C and S6). Astrocytes within perfused vascularized regions, by contrast, did not express VEGF-A, as would be expected given their access to circulating oxygen. Together, these results show that astrocytic VEGF-A immunoreactivity is bidirectionally regulated by tissue oxygen levels – a finding that strongly supports specificity of the antibody.

We also tried a low-O_2 experiment, in which animals were exposed to 10% oxygen from P0-2. Unfortunately we were unable to draw conclusions about astrocyte VEGF-A expression in low-O2 mice, because hyaloid vasculature failed to regress in these animals and was strongly VEGF-A immunoreactive, interfering with our astrocyte imaging. However, based on the results of the experiment noted above, we were still able to conclude that (relative) hypoxia upregulates VEGF-A expression (Fig. S4C).

Second, We have added new images to more clearly document the VEGF-A immunostaining pattern obtained with this antibody in normal, untreated mice. These figures (newly modified Fig. 7A and new Supplemental Fig. S4A,B) clearly show that VEGF-A immunostaining recapitulates the staining pattern previously documented for *Vegfa* mRNA in developing mouse retina (e.g. West et al., 2005 PMID 15790963; Gerhardt et al., 2003 PMID 12810700).

There are two features of this staining pattern that go towards addressing the specificity issue raised by the reviewer. First, our antibody staining confirms the previous conclusion that *Vegfa* mRNA is selectively expressed by astrocytes. Second, antibody staining confirms the previous finding that *Vegfa* mRNA expression levels are regulated by access to vasculature. Both mRNA and protein are highly expressed by astrocytes of hypoxic avascular retina, whereas astrocytes in perfused central regions express minimal VEGF-A mRNA or protein (see West et al., 2005 & Gerhardt et al., 2003 for mRNA and Figs. 7A, S4C, and S6 for protein). The concordance of mRNA and protein staining lends confidence that the antibody does indeed recognize VEGF-A.

Moreover, these stains provide a particularly compelling case for antibody specificity because they are internally controlled: VEGF-A immunoreactivity varies *within the same retina* based on the hypoxic status of astrocytes (Fig. 7A; Supplemental Figs. S4C and S6). This finding rules out any possibility that staining levels were altered by differences in sample preparation or staining efficacy between tissues, leaving oxygen availability as the most likely determinant of staining levels.

The concordance between previous stainings and our own immunohistochemical results are now noted in the Results (lines 409-418).

Third, we now draw on prior literature to support the specificity of this VEGF-A antibody. As we now note in the revised Methods (lines 172-177), other labs have previously validated it and have used it for similar purposes. For example, the antibody has been used in other tissues to read out increases or decreases in HIF pathway activity: Immunoreactivity was increased in *Vhl* KO mutant

© 2021. Published by The Company of Biologists under the terms of the Creative Commons Attribution License (https://creativecommons.org/licenses/by/4.0/).

mice that stimulated HIF signaling, and was decreased in Hif1□ KO mutant mice that blocked HIF signaling (Blouw et al., 2007; PMID 17297464). These results support our use of the antibody to identify HIF-deficient cells.

The antibody has also been used to assess VEGF-A expression in developing mouse retina. Morita et al. (2017; PMID 28033674) compared the antibody side-by-side with *Vegfa* in situ hybridization, showing that both methods of VEGF-A detection yield similar staining patterns – both of which are similar to our Fig. 7A. Further, both staining methods demonstrated that astrocytes upregulate VEGF-A expression under hypoxic conditions (Morita et al., 2017).

Altogether, these results – together with our own newly added data – demonstrate that the antibody is overwhelmingly likely to be specific for VEGF-A, since it shows the same astrocyte cellular specificity and oxygen regulation as the *Vegfa* transcript.

1.6)*5.Following up on point 4, it is also possible that astrocytes bind VEGFA that has been produced by other cells, which means that there is not necessarily a correlation between VEGFA IHC positive astrocytes and hypoxic astrocytes.*

To investigate the cellular source of VEGF-A protein, we performed new VEGF-A staining studies, focusing on subcellular localization (Supplemental Fig. S4A,B). This analysis demonstrates that the antibody is very effective at detecting intracellular VEGF- A – presumably within the secretory pathway – but it is not particularly effective at detecting extracellular secreted VEGF-A. Co-staining with the astrocyte cell-surface marker PDGFR□ clearly shows that VEGF-A immunoreactivity is intracellular, and rarely labels astrocyte processes (Supplemental Fig. S4A,B). Furthermore, the subcellular staining pattern obtained with this antibody is typical of secretory pathway organelles such as endoplasmic reticulum or Golgi apparatus.

Based on this expression pattern, we conclude that the antibody highlights cells that are producing and secreting VEGF-A, but not those that accumulate extracellular VEGF-A. This finding supports our use of VEGF-A immunostaining as a cell- autonomous readout of HIF signaling status.

1.7)*What is the proportion of VEGFA negative astrocytes in the AC-Hif2a KOs (in comparison to all astrocytes)?*

To address this point we have added a graph showing these data for P2 AC-Hif2□- KO mutants and littermate controls (Supplemental Fig. S5B).
At P8-10, when mutants can be classified as VEGF-high or VEGF-low, it did not make sense to quantify this proportion since virtually all astrocytes are either VEGF- positive or -negative in these two types of mutants. However, to give the reader a better sense of the extent of VEGF-A expression in VEGF-low mutants, we have now added example whole retina VEGF-A images (Supplemental Fig. S6). These images show that a small number of VEGF-A^{+} cells can sometimes remain in the mutant nerve fiber layer, although the vast majority of astrocytes are VEGF-A negative.

1.8)*7.The image in Fig. 7B is not convincing at all. The background on the left side of the VEGF-A panel is clearly much darker, which also the location where all the VEGFA negative astrocytes happen to be.*

We apologize for the poor quality of the image. For the new version of this figure - now Fig. 7C - we have z-projected a larger volume of the confocal stack which should address the reviewer's concern. As is now clear, the cells on the left side of the image are undoubtedly VEGF-A negative. To increase confidence in our ability to identify double-labeled cells, we have also improved the image quality for other VEGF-A/Sox9 double staining panels - e.g. Fig. 7A and Fig. 9 (formerly Fig. 8). Also, the new images in Supplemental Fig. S4A,B further illustrate the reliability of our double labeling assessments. When one considers the complete set of VEGF-A/Sox9 double-staining images included throughout the revised manuscript, it should be evident that the double labeling method works quite nicely and leaves little doubt as to whether a particular Sox9+ cell is also VEGF-A+.

1.9)*Also, at what location (in relation to the vessels) where the measurements shown in Fig. 7 C*

© 2021. Published by The Company of Biologists under the terms of the Creative Commons Attribution License (https://creativecommons.org/licenses/by/4.0/).

taken?

The measurements in the former Fig. 7C (now Fig. 7D) were from both vascular and avascular regions – we sampled uniformly across eccentricities as described in the Methods (lines 184-185). The use of both vascular and avascular retina is now noted in the legend to Fig. 7.

We saw no reason to restrict our analysis to one retinal location because, in wild- type animals, most astrocytes are VEGF-A$^+$ at P2 regardless of whether vessels are present. As we write in the Results: "Even though VEGF-A was downregulated in vascularized central retina (Fig. 7A), expression was not yet entirely extinguished – a similar fraction of P2 astrocytes were VEGF$^+$ regardless of retinal location (88.7% in vascular central retina; 94.8% in avascular regions" (lines 437-439). Therefore, retinal location should have little impact on the outcome of this analysis.

1.10) *8.I am intrigued by the VEGFA stain in the "VEGF-low" AC-Hif2a KOs (Fig. 7 D). If there are no vessels, we can assume the retina to be fairly hypoxic and RGCs ought to express VEGFA strongly, why is this not picked up by the VEGFA stain?*

At the reviewer's suggestion we have revisited our VEGF-A staining in VEGF-low mutants and we have not observed any instances of immunoreactivity within the ganglion cell layer. We cannot say definitively why the RGCs do not express VEGF-A. One possibility is that the hyaloid vessels, which persist in Hif2a mutants, are able to supply sufficient oxygen to prevent HIF activation in RGCs. Whether or not this is the full explanation, we feel it is clearly beyond the scope of this paper to investigate why RGCs do not express VEGF-A in these mutants.

If the reviewer's concern is more related to the reliability of the VEGF-A antibody, we have added substantial new data supporting this antibody's specificity and suitability for our studies (see point 1.5 above).

1.11) *9.The authors have shown profound GFAP upregulation in Muller cells (Fig. 5). In the AC-Hif2a KO background this should lead to VEGFA deletion also in Muller cells.*
Have the authors considered the confounding effects that might cause?

It is certainly possible that Hif2¤ might become deleted in Müller cells, as the reviewer suggests. We have now added a sentence to the Discussion to acknowledge this possibility (lines 568-570). But we think this is unlikely to have a major effect on our results or interpretation, for the following two reasons:
First, based on our analysis of published single-cell RNA-seq data (Clark et al. 2019; PMID 31128945), Müller glia do not appear to express the *Epas1* gene (i.e. Hif2¤ gene) at appreciable levels. Instead, the HIF pathway is likely driven by HIF1¤ in these cells. Therefore deletion of the HIF2¤ gene in Müller cells is unlikely to have a major phenotypic effect.
Second, even if Hif2¤ were to become expressed by Müller cells in NOIR mice, its deletion would likely occur too late to influence astrocyte proliferation. In the NOIR paradigm, the peak of astrocyte proliferation is P6; this leads to increases in cell number by P8. By contrast, we did not observe GFAP upregulation in Müller glia until P12.
Therefore, enhanced GFAP expression (and any resulting expression of Cre recombinase) occurs subsequent to the major hypoxia-induced proliferation events that we have studied in this paper. These two factors lead us to conclude that cell-nonautonomous effects driven by Müller cells are unlikely to contribute significantly to the astrocyte proliferation phenotype in AC-HIF2¤-KO mice. By contrast, the data in Fig 7A-D clearly show a cell autonomous function for HIF2¤ within astrocytes – it is autonomously required for VEGF-A expression and astrocyte proliferation. Therefore, even if a minor Müller-driven component of the phenotype were present, this would not substantially alter our conclusions.

1.12) *10.What are the outcomes for retinal vascularisation in the hyperoxia exposed AC-Hif2a KO mice?*

In the revised manuscript we now show the retinal vascular phenotype in both normoxic and NOIR-exposed mutants (new Fig. 8; new Supplemental Fig. S6). There are two key takeaways from this new analysis:

© 2021. Published by The Company of Biologists under the terms of the Creative Commons Attribution License (https://creativecommons.org/licenses/by/4.0/).

First: VEGF-low mutants, regardless of exposure to hyperoxia, entirely lack vessels. This is now noted in the Results (lines 455-458) and the Discussion (lines 571-575 and 660-662).

Second: Even in normoxia, VEGF-high mutants often show a delay in angiogenesis (Fig. 8B; Results, lines 462-464). This finding supports our conclusion that these animals are not entirely normal – the onset of angiogenesis may be delayed during the period when HIF+ astrocytes are outcompeting mutant astrocytes to take over the retina. We comment on this new evidence supporting the "takeover" model in the Discussion (lines 579-588).

Minor

1.13) *11.What is the p value for the linear regression model of Fig4C?*

The P value ($P = 0.0004$) has been added to Fig. 4. There is a significant deviation from slope = 0.

1.14) *12.The terms of hyperoxia and hypoxia can be confusing to non-specialist readers. So, whilst mice are exposed to hyperoxia, when they are returned to room air, the retinal astrocytes experience hypoxia.*

We thank the reviewer for pointing this out – we think changing this will vastly improve clarity for the reader. Throughout the text and figure labels, most instances of "hyperoxia" have been changed to "NOIR paradigm," or "high-O2", or variants thereof.

Reviewer #1 (Significance (Required)):
1.15) *The study is interesting and potentially useful, but at this stage not entirely convincing.*

We are glad the reviewer finds the story interesting. As described above, we have made numerous changes to fortify and strengthen the data supporting our conclusions. We trust that this will serve to alleviate the reviewer's concerns and to make our conclusions more convincing.

Reviewer #2 (Evidence, reproducibility and clarity (Required)):

2.1)*The authors explored the effect of hyperoxygen exposure on glial and vascular development. They also analyzed the phenotype in astrocyte-specific Hif2 knockout mice. The conclusion is that astrocyte oxygen sensing through Hif2 regulates the number of astrocytes required for proper vascular development. Overall, the data are of good quality and convincing.*

We are glad the reviewer found the data to be convincing.

Reviewer #2 (Significance (Required)):
2.2) *However, it's hard to identify any novel finding upon previous papers (Morita et al., Birth Defects Res B Dev Reprod Toxicol. 2016* [PMID 27792858]*; Duan et al., Plos One 2014* [PMID 24475033]*; Duan et al., Sci Rep 2017* [PMID 20452322]*). The present study looks just a replication of previous ones listed above, although there are differences in mouse strains and in the severity of retinal degeneration.*

In our original Discussion we specifically addressed each of these three papers and how our work relates to them. We regret that these passages were evidently not clear enough for the reviewer to see that our paper makes distinct contributions. We have revised our Discussion in numerous places to more clearly emphasize the novelty and significance of our work.

We strongly disagree with the idea that our study is a mere replication of these three papers. Neither these papers, nor any other previous paper, has ever reported the central finding of our study – i.e. that hypoxia drives retinal astrocyte proliferation.
Moreover, we show that hypoxia disrupts astrocyte pattern formation, and that these hypoxia-induced astrocyte phenotypes are linked to developmental vasculopathies reminiscent of human disease. None of these findings were reported in the papers cited by the reviewer (or, to our knowledge, in any other paper).

© 2021. Published by The Company of Biologists under the terms of the Creative Commons Attribution License (https://creativecommons.org/licenses/by/4.0/).

Based on our new results, our paper reaches a key central conclusion that, to our knowledge, is unique to our paper: We identify astrocyte overproliferation as a mechanism by which astrocytes could drive oxygen-induced retinopathy. This contribution is highly significant because it answers a long-standing question in the field: While it had long been speculated that astrocytes could have a pathogenic role in retinopathy of prematurity (ROP), or in the animal models of this human disease, it was unclear whether or how this actually occurs. Our results suggest that hypoxia-induced astrocyte overproliferation may be an important pathogenic mechanism, raising the possibility that such proliferation could be targeted therapeutically to ameliorate disease. These important new ideas and concepts, which we spell out in detail in our Discussion, are never brought up in any of the three papers cited by the reviewer. Nor would it make sense for those authors to have done so, because their papers were aimed at different purposes.

Of course there are some similarities between these papers and ours, because we drew on past work in designing our studies. For example, one of the reasons we decided to study astrocyte-specific Hif2□ knockout (AC-Hif2□-KO) mice in the NOIR model was that Duan et al (2014) had previously shown a related phenotype for these mice in normoxia. So it made sense to use the same critical reagent as the previous paper to address our own unique question related to oxygen-induced pathology.

One of the most important reasons for similarity with the cited papers was that there were conflicting results in the literature, which we needed to resolve in order to perform the new parts of our study. Indeed, one of the major contributions of our paper is that it sorts out two such conflicts, which were a source of confusion for the field.

1. In 2016, two papers reported the phenotypic effects of early oxygen exposure in mice. Morita et al. (2016; cited by the reviewer) found that vascular defects in a NOIR- like model are fairly minor and that they are transient – errors are corrected by P20. By contrast, a contemporaneous paper by McMenamin et al. (2016; PMID 27918825) came to the opposite conclusion, finding that early O2 exposure causes long-lasting vascular pathology. Our work is important because it helps to clarify the effects of neonatal O2 exposure and to resolve the underlying causes of vascular defects in these models (see Introduction, lines 96-98).

2. Two previous papers examined the retinal phenotype of AC-Hif2□-KO mice reared in normoxia. Weidemann et al. (2010; PMID 20544853) did not find any retinal phenotype in these mice, whereas Duan et al. (2014; cited by the reviewer) reported absence of retinal vasculature and reduced numbers of astrocytes. The conflicting reports as to the role of astrocytic HIF signaling was a major source of confusion and debate within our field. Our study is important because it not only replicates the Duan et al. result, but it also provides an explanation for why the results of the original two studies were different: We show that if Cre recombinase does not delete Hif2□ in a substantial fraction of astrocytes, the remaining wild-type ones can take over the retinal astrocyte population to support blood vessel development. This key contribution is highlighted in the Discussion (lines 543-592).

Despite these similarities, there are key differences between our paper and those cited by the reviewer – each of which goes towards the major points that we highlighted at the start of this section. To summarize the key differences:

Morita et al., 2016: This paper used an oxygen exposure paradigm very similar to our NOIR paradigm. But they did not identify long-lasting vascular pathologies, nor did they study the effects of oxygen exposure on astrocyte proliferation.

Duan et al., 2014: This paper studied astrocyte-specific Hif2□ knockout mice. But unlike our paper:

- They did not study the role of astrocytic HIF signaling in the context of neonatal oxygen induced retinopathy.
- They concluded that Hif2□ is dispensable for astrocyte proliferation, based on a BrdU experiment. However, we show that this conclusion was likely incorrect, due to variable Hif2□ deletion by the Cre driver line. Using VEGF-A labeling to establish the HIF signaling status in each astrocyte, we revealed that Hif2□ does indeed have a role in astrocyte proliferation.

© 2021. Published by The Company of Biologists under the terms of the Creative Commons Attribution License (https://creativecommons.org/licenses/by/4.0/).

Duan et al., 2017: This paper examined retinal astrocyte development in mutant mice lacking blood vessels, due to an endothelial cell-specific deletion of *Vegfr2*.

- The authors focused on showing that vasculature serves to drive astrocyte differentiation. Thus, most of this paper is tangential to our study.
- Proliferation was not addressed, except for one supplemental figure. Even in this figure the authors did not test whether hypoxia drives astrocyte proliferation.
- There is an oxygen-exposure experiment but their paradigm (75% O2 for one day, from P0-P1) is completely different to what we used and was not designed to study vascular pathology. Astrocyte proliferation was not investigated.
- There is an experiment with AC- Hif2¤-KO mice but it is unrelated to our study.

We have revised our Discussion to more clearly emphasize the features that distinguish our work, incorporating many of the points made here.

Reviewer #3 (Evidence, reproducibility and clarity (Required)):

Summary:

3.1) *The study by Perelli et al. explores the role of hypoxia-driven astrocyte proliferation in mediation of pathological angiogenesis during retinopathy of prematurity. The authors examine CD1 neonatal pups exposed to 75% oxygen from P0-P4, followed by analyses of the development of the astrocyte network and the vasculature during the subsequent relative hypoxia period upon return to normoxia. The role of HIF2a in regulating VEGFA expression in astrocytes is examined using GFAP-Cre; Hif2a mice. Overall, this is an interesting study but very importantly, the genetic background of the mice and the establishment of vascular pathology have not been properly explained.*

We are glad the reviewer found the study interesting. The revised manuscript addresses the issues of genetic background and vascular pathology – please see below for details, which are provided in response to the reviewer's specific comments.

Major comments

3.1)*1.The mouse genotype contributes very decisively to establishment at least of the vascular phenotype in retinopathy. The absolute majority of vascular analyses as a consequence of OIR is done using C57Bl/6. In some strains such as FVB or Balb/c, pathological angiogenesis is not seen in response to the P7-P12 regiment (Ritter et al., J Clin Invest. 2006 Dec 1; 116(12): 3266-3276). See for example O´Bryhim et al., Mol Vis. 2012; 18: 377-389 with regard to impact of the mouse strain genetic background on avascularity in response to hyperoxia. Therefore, it is very important that the authors describe and are consistent, with regard to the strains they use in this study. Most of the analyses appears to have been done using CD1. Why was this strain chosen, and not C57? This should be motivated. How does vascular development {plus minus} hyperoxia compare between DC-1 and C57?*

The reviewer raises a very important issue concerning mouse strains. Our motivation for using CD-1 was that it had previously been used in the Morita et al (2016) study cited above (PMID 27792858). We wanted to be able to compare our results from the P0-P4 hyperoxia regime to their previously published results. We also chose CD-1 because timed-pregnant dams are readily available, making it an ideal strain for assembling large multi-litter cohorts of age-matched mice as required by our experimental design (see point 1.2 above).

However, we agree with the reviewer that it is important to show that CD-1 results generalize to the much more commonly-used C57Bl/6 strain. For that reason we have now performed additional studies using C57Bl/6J mice obtained from Jackson Labs (Results, lines 279-283 and 298-300). As shown in the newly-added Supplemental Fig. S3, C57Bl/6J mice exposed to the NOIR protocol show the same vascular and astrocyte phenotypes as NOIR-exposed CD-1 mice. Specifically, both strains show 1) delayed and mispatterned angiogenesis; 2) reverse angiogenic wavefront originating from the retinal

© 2021. Published by The Company of Biologists under the terms of the Creative Commons Attribution License (https://creativecommons.org/licenses/by/4.0/).

periphery; and 3) excess astrocyte numbers arising from elevated mitotic activity.

3.2)*The GFAP-Cre mice appear to be on FVB background at Jax (FVB-Tg(GFAP- cre)25Mes/J Stock No: 004600). Did the authors backcross these mice to CD-1 or use a mixed background? The Epas1floxed mice are in the SvJ background at Jax. The genetic backgrounds of the different strains are not described in the methods and the potential impact different genetics may have on the results is not considered. That is absolutely required.*

The genetic background of our mutant strains is indeed crucial information that should have been included in the original manuscript. This information has now been added to the Methods (lines 120-124).
As noted in the revised manuscript, the GFAP-Cre strain was maintained in our colony by backcrossing to C57Bl/6J. We maintained the mice in this way for ~ 3 years (at least 8 generations) prior to introducing the Cre line into the Hif2a-flox strain. Hif2a- flox mice obtained from Jax were on a mixed 129X1-SvJ-C57Bl/6J background. They remained on a mixed background for these studies, with additional crosses to the (largely) C57Bl/6J GFAP-Cre strain.
Strain-matched controls (i.e. Hif2a^{WT} mice from the mixed 129X1-SvJ-C57Bl/6J strain) were used for all mutant studies. When these Hif2a^{WT} mice were exposed to the NOIR protocol, we did not find any obvious differences in vascular phenotypes compared to C57Bl/6J or CD-1. This information is now noted in the Results (lines 481- 483).

3.3)*Also, I lack information on when and how tamoxifen was delivered and the extent of deletion. All pups should have received tamoxifen - this is essential information which is not given.*

Tamoxifen was not used in this study. The GFAP-Cre line used here is not tamoxifen inducible – that is, it does not require tamoxifen to induce recombination. We regret any lack of clarity in our text that led to the reviewer’s confusion on this point.

3.4)*Moreover, as it is known that the establishment of the eye pathology after high- oxygen is highly dependent on pup weight at the point of evaluation, this information must be given in order to clarify ascertain that the authors are studying a defect specific to retinal pathology and not due to a general developmental defect.*

The reviewer has asked us to address whether the NOIR-induced astrocyte and vascular phenotypes are specific – i.e., does oxygen affect the *specific* developmental programs that generate these two cell types? Or does the NOIR paradigm have *general* effects on animal growth and/or eye development? Unfortunately we did not measure pup weight while doing these experiments, so we cannot provide the exact data requested by the reviewer without repeating our entire study from scratch. Instead, as an alternative approach to assuage the reviewer’s concerns, we measured the size of the retina across development in control and NOIR mice (see Results, lines 276-278, and newly added Supplemental Fig. S2). Overall growth of the retina can be viewed as a general developmental phenomenon. Thus, if the NOIR protocol causes general eye development defects, we would expect that growth of the retina should be impaired – particularly during the period when astrocyte and vascular phenotypes emerge (i.e. P4- P12). By contrast, if NOIR causes specific defects in astrocyte/vascular development, we would expect retinal growth to be unaffected by NOIR exposure.

Consistent with the idea of specific astrocyte/vascular phenotypes, we found that retinal size was not significantly different between NOIR and control groups (Supplemental Fig. S2). We observed a small rightward shift of the size curve for oxygen-exposed animals relative to their normoxic littermates, but this difference was present already at P4, when the animals exited the oxygen chamber. Subsequently, during the critical P4-P12 period when astrocytes and vessels become disturbed, eye size increased at similar rates in both NOIR and normoxic animals (Supplemental Fig. S2 – note similar slopes of the NOIR and normoxia plots). Based on these findings it seems highly unlikely that overall growth retardation could contribute to the phenotypes we report here. Instead, we conclude that the astrocyte and vascular phenotypes are most likely to be caused by specific effects of oxygen on their development.

A previous study of P0-P4 hyperoxia-exposed animals reached a similar conclusion (Morita et al., 2016; PMID 27792858). When these authors measured pup weight across development, they found a

© 2021. Published by The Company of Biologists under the terms of the Creative Commons Attribution License (https://creativecommons.org/licenses/by/4.0/).

growth pattern that was strikingly similar to our retina size measurements: At the end of the high-O_2 period, treated animals were slightly underweight; but after return to room air their weight increased at a similar rate as normoxic controls. Thus, regardless of whether the readout is retina size or animal weight, the conclusion is the same: The stress of relative hypoxia upon return to room air has minimal effects on general features of mouse development or eye growth.

3.5)2.*The authors make the point that developmental formation of the astrocytic network is a prerequisite for vascular development and that excess proliferation of astrocytes in hypoxia (relative or true) may drive the vascular pathologies seen in eye diseases such as retinopathy of prematurity. In Fig. 1, the authors show that no vessels are formed in the CD1 strain exposed to hyperoxia during P0-P4. When hyperoxia occurs during P2-P4, a large avascular area is formed at P8 similar to the "traditional" finding, meaning in C57 mice, I assume? Please explain [….]*

Yes, this sentence was referring to the standard P7-P12 OIR paradigm which is performed in C57Bl/6 mice.

The fact that different strains are used in our experiment vs. standard OIR does not invalidate the point we were making in this part of the manuscript: There are clear similarities between the phenotype shown in Supplemental Fig. S1 and standard OIR. In both cases, mice were exposed to high oxygen after the onset of angiogenesis. And in both cases, substantial vasculature in central retina is lost by the end of the high-O_2 period.

While the reviewer is correct that there are key strain differences related to other OIR phenotypes – particularly neovascular tuft formation – vaso-obliteration during the high-O_2 phase (P7-P12) is not specific to the C57Bl/6 strain. This fact is validated by the very same papers cited above by the reviewer (Ritter et al., 2006 PMID 17111048; O'Bryhim et al., 2012 PMID 22355249; see point 3.1 above), and is confirmed by a third paper (Dorrell et al., 2010 PMID 19544395). All three of these studies compared the extent of avascularity at P12 in OIR-exposed C57Bl/6 vs. Balb/c mice. And all three papers concluded that O_2-induced vessel loss was comparable in both strains.

Our assertion about similarities between models was limited to the vaso-obliterative effects of high oxygen. It is well known that different strains of mice respond differently to the hypoxic phase of standard OIR, which begins upon return to room air at P12.
However, based on the papers cited above, we are entirely justified in pointing out that CD-1 mice treated with high oxygen from P2-P4 or P4-P8 show similar vessel loss to the "traditional" OIR model – regardless of which strain is used for traditional OIR.

3.6)*[…] The statement on p18 concerning "Starting hyperoxia at P0 has qualitatively different effects compared to later stage" with a reference to Louis Smith who uses C57, is not valid.*

Our overall conclusion here – that there are qualitative differences when high-O_2 is started at P0 vs later ages – is supported by our own data. The citation was not needed to support this point. Our data in Fig. 1 and Supplemental Fig. S1 show a clear qualitative difference: When O2 is started at P0, angiogenesis is blocked; by contrast, when O2 is started later, angiogenesis proceeds normally although there is a loss of vessels that were formed prior to onset of O2 treatment.

The purpose of the citation was not to bolster our conclusion, but rather to point out that the vaso-obliteration we observed in Fig. S1 resembles that seen in OIR models. (As we detail in point 3.5 above, there are clear similarities between the two situations). To address the reviewer's concern we have changed the citation. Rather than citing the C57Bl/6 work (Smith et al., 1994), we now cite the three papers listed above in point 3.5, which demonstrate similar extent of vaso-obliteration in multiple strains (Results, line 252).

3.7)*[…] However, the effect of hypoxia-regulated VEGFA induction in response to the relative hypoxia after high O2 incubation, is in formation of pathological angiogenesis, so-called vascular tufts. Did vascular tufts become established in the CD1 strain after the "conventional" P7-12 exposure and after the P0-P4 exposure? If such tufts are not formed, conclusions on the development of the astrocytic network preceding the establishment of any hypoxia/VEGFA-dependent vascular pathologies cannot be made.*

© 2021. Published by The Company of Biologists under the terms of the Creative Commons Attribution License (https://creativecommons.org/licenses/by/4.0/).

Our conclusion that astrocyte network errors precede vascular pathologies is based on the simple fact that astrocyte phenotypes are present prior to angiogenesis (Fig. 4). In normal development, it has been known for 25 years that vasculature grows over a pre-existing astrocyte network. Here we show that this is also the case in NOIR mice.

Fig. 4A and 4B illustrate how the angiogenic wavefront grows over a pre-existing array of astrocytes and their arbors, both in normoxia (bottom panels) and in NOIR-exposed mice (middle panels). These data, in our view, are sufficient to support our contention that astrocyte network disruptions precede the establishment of vascular pathology – indeed, they entirely precede the formation of vasculature.

It is true, as the reviewer states, that neovascular tufts are the key pathology that manifests in the traditional OIR model. By contrast, in NOIR mice we find a range of distinct vascular pathologies – these are documented in Fig. 2 (for CD-1 mice) and Supplemental Fig. S3 (for C57Bl/6J mice). Some of the NOIR pathology may be tuft-like (see newly added Fig. 2D, middle panel), although these are clearly not the same as the classic neoangiogenic tufts seen in traditional OIR. Regardless of whether they can be defined as tufts, we would argue that this point has little bearing on the issue raised by the reviewer about the relative order of astrocyte/vascular defects. Because astrocyte number and patterning are disrupted prior to arrival of the angiogenic wavefront, our statement that astrocyte errors come first is valid.

The reviewer asks whether CD-1 mice develop vascular tufts in the conventional P7-P12 OIR model. We have not examined this issue. Such experiments are clearly outside the scope of our study, which was focused solely on neonatal oxygen exposure.

It seems that the reviewer's larger point is again directed towards strain effects. Formation of neovascular tufts in conventional OIR is highly strain-dependent – only C57Bl/6 does so reliably. If the reviewer is worried that this strain difference might extend to the NOIR paradigm, we would make two points to assuage this concern. First, the vascular phenotypes induced by the NOIR protocol (see revised Fig. 2) do not include the kind of neovascular tufts that are subject to strain effects in standard OIR. Second, our newly-added data from C57Bl/6 mice (Supplemental Fig. S3) demonstrate that both CD-1 and C57Bl/6 strains respond similarly in the NOIR paradigm.

3.8)*3.In Fig. 1B, dense IB4-positive patches are seen at P12. Are these tufts or sheets of ECs? This should be better described and shown at high magnification. It does not seem to resemble the peripheral sheets of endothelial cells highlighted in Fig. 2D.*

To address this important point raised by the reviewer, we now provide a much more detailed description of the vascular phenotypes (see Results, lines 255-279). We have also added a high magnification image of the dense patches as requested by the reviewer (Fig. 2D, middle panel). These dense regions do not resemble the classic vascular tufts that typify standard OIR pathology – they are too small. However they do have some tuft-like qualities: they appear to be regions where the vasculature has failed to grow two-dimensionally along the nerve fiber layer but instead has formed a clump or an adhesion with the lens/vitreous. This anatomy is now described in the legend to Fig. 2.

3.9)*4.On page 18, the authors state that 50% of the hyperoxia-treated mice had persistent pathology. I assume this is in the wildtype CD1 mice and unrelated to efficiency of floxing of Hif2a. This variable establishment of pathologies and which one may or may not be established, is not clear from the results except for the hemorrhaging. What is meant here with "persistent"? Please overall explain and present the vascular and astrocytic pathology and the penetrance in a clear manner.*

As noted in Point 1.1 above, we now provide much more detailed information as to the penetrance of the various vascular and astrocytic phenotypes. Please see revised Results (lines 255-268 & 333-340) and legends to Figs 2 and 4.

Minor
3.10) *5.In Fig 1 C, please make it clear which hyperoxia period the mice were exposed to.*

© 2021. Published by The Company of Biologists under the terms of the Creative Commons Attribution License (https://creativecommons.org/licenses/by/4.0/).

We have changed the label to specify the P0-4 regimen.

3.11) *6.Data in Fig 5 needs to be quantified.*

We now specify the frequency with which we observed each of the anatomical phenotypes noted in Fig. 5. See Results, lines 365-377.

3.12) *7.The authors could try to immunostain for HIF2 to classify Cre+ mice as successfully deleted?*

We did attempt to stain for HIF2 but unfortunately the antibody did not work for immunohistochemistry. That is why we turned to the VEGF-A antibody. In response to critiques from Reviewer 1, we have now performed a far more extensive validation of this antibody which should help to assuage some of this reviewer's concerns (see points 1.5 and 1.6 above). These data are included in the revised Fig. 7 and the new Supplementary Fig. S4.

3.13) *Reviewer #3 (Significance (Required)):*

The role of VEGFA produced by astrocyte for vascular development is well established, however much less is known about a potential driving role of astrocytes in vascular pathologies such as in retinopathy. I therefore think this study would be of broad interest.

We thank the reviewer for the kind words about the potential broad appeal of this work.

Submission to Development

First decision letter

MS ID#: DEVELOP/2021/199418

MS TITLE: Environmental Oxygen Regulates Astrocyte Proliferation to Guide Angiogenesis during Retinal Development

AUTHORS: Robin M Perelli, Matthew L O'Sullivan, Samantha Zarnick, and Jeremy N Kay

You will be pleased to hear that the referees are happy with your revisions and recommend publication of your manuscript. The referees' comments are appended below, or you can access them online: please go to BenchPress and click on the 'Manuscripts with Decisions' queue in the Author Area.

Prior to proceeding to publication, could you please check that the word count of your manuscript meets the guidelines for Development (as you can see below this is 7000 words but the maximum can be stretched to about 7500 words). For some reason Benchpress did notautomatically calculate the word count but we estimate that the manuscript is currently over the limit.

Reviewer 1

Advance summary and potential significance to field

The role of VEGFA produced by astrocyte for vascular development is well established, however much less is known about a potential driving role of astrocytes in vascular pathologies such as in retinopathy. I therefore think this study is an important contribution of broad interest.

© 2021. Published by The Company of Biologists under the terms of the Creative Commons Attribution License (https://creativecommons.org/licenses/by/4.0/).

Comments for the author

The authors have performed a very ambitious revision and responded fully to my questions. I have no further comments/criticisms but regard the study as an important, scientifically sound contribution.

Reviewer 2

Advance summary and potential significance to field

In this study Perelli et al. demonstrated that exposure of mouse pups to hyperoxia from P0-P4, followed by returning to normoxia leads to overproliferation of retinal astrocytes, which appears to impact on retinal vascularization. The authors also showed that in astrocyte specific HIF2a KO mice the retinal astrocytes are present in reduced numbers, do not over proliferate in the P0-P4 hyperoxia model and show reduced VEGF expression. This is associated with a lack of retinal vascularization. Overall, this is a well executed study and well presented manuscript. The findings are very interesting and providing novel insights about retinal vasculature development.

Comments for the author

I have reviewed a previous version of this manuscript Review Commons EMBO platform and the authors have addressed all my previous concerns. I have therefore only one comment to add here. The authors might want to include in their discussion (Intrinsic astrocyte hypoxia… section) the finding by Scott et al. 2010 (20686684), who reported only minor impact on vascular development after astrocyte specific VEGF-A deletion.

First revision

Author response to reviewers' comments

We thank the reviewers for their kind comments and are very pleased that they favor publication of our manuscript.

Second decision letter

MS ID#: DEVELOP/2021/199418

MS TITLE: Environmental Oxygen Regulates Astrocyte Proliferation to Guide Angiogenesis during Retinal Development

AUTHORS: Robin M Perelli, Matthew L O'Sullivan, Samantha Zarnick, and Jeremy N Kay
ARTICLE TYPE: Research Article

Apologies for the delay in responding to your manuscript submission. I am happy to tell you that your manuscript has been accepted for publication in Development, pending our standard ethics checks.

© 2021. Published by The Company of Biologists under the terms of the Creative Commons Attribution License (https://creativecommons.org/licenses/by/4.0/).
